# Triciribine increases LDLR expression and LDL uptake through stabilization of *LDLR* mRNA

**DOI:** 10.1038/s41598-018-34237-6

**Published:** 2018-11-01

**Authors:** Katrine Bjune, Lene Wierød, Soheil Naderi

**Affiliations:** Unit for Cardiac and Cardiovascular Genetics, Department of Medical Genetics, Oslo University Hospital, Oslo, Norway

## Abstract

Low-density lipoprotein receptor (LDLR) is a key regulator of the metabolism of plasma low-density lipoprotein cholesterol (LDL-C), the elevated levels of which are associated with an increased risk of cardiovascular disease. Therefore, enhancing LDLR expression represents a potent treatment strategy for hypercholesterolemia. Here, we report that in cultured human hepatoma cells, triciribine, a highly selective AKT inhibitor, increases the stability of *LDLR* mRNA, an event that translates into upregulation of cell-surface LDLR levels and induction of cellular LDL uptake. This effect of triciribine requires ERK activity and is partially dependent on the intervening sequence between the AU-rich elements ARE3 and ARE4 in *LDLR* 3′UTR. We also show that triciribine downregulates the expression of *PCSK9* mRNA and blunts the secretion of its protein. Notably, triciribine was found to potentiate the effect of mevastatin on LDLR protein levels and activity. We also show that primary human hepatocytes respond to triciribine by increasing the expression of LDLR. Furthermore, a pilot experiment with mice revealed that a two-weeks treatment with triciribine significantly induced the hepatic expression of LDLR protein. These results identify triciribine as a novel LDLR-elevating agent and warrant further examination of its potential as a hypocholesterolemic drug either as monotherapy or in combination with statins.

## Introduction

Elevated plasma low-density lipoprotein cholesterol (LDL-C) is associated with the risk of developing atherosclerotic cardiovascular disease (ASCVD), a condition that accounts for a large proportion of deaths worldwide^1–3^. Therefore, lowering of plasma LDL-C levels is the most effective method to prevent development of ASCVD. Low-density lipoprotein receptor (LDLR) is an integral membrane protein that is most abundantly expressed in the liver, where it binds to and removes LDL-C from the circulation by endocytosis^4,5^. Therefore, the amount of LDLR expressed in hepatocytes inversely correlates with the level of plasma LDL-C.

LDLR levels are controlled by a multi-layered regulatory mechanism: At the transcriptional level, *LDLR* gene expression is controlled by a cholesterol-responsive negative feedback mechanism through sterol regulatory element-binding protein-2 (SREBP-2)^6^. Post-translational regulation of LDLR is primarily governed by PCSK9 that upon binding to cell-surface LDLR mediates its degradation^7^. The post-transcriptional regulation of LDLR is mainly achieved through modulation of its mRNA stability^8–11^.

*LDLR* mRNA is a labile transcript that contains a 2.5 kb-long stretch of 3′ untranslated region (3′UTR)^9,12^. Embedded within the *LDLR* 3′UTR are several *cis*-regulating elements that modulate the rate of *LDLR* mRNA degradation through their interaction with *trans*-regulatory RNA binding proteins^8,13^. Certain *trans*-regulating factors act as mRNA stabilizers, while others promote mRNA decay^14,15^. Both types of factors have been reported to interact with *LDLR* mRNA. For instance, *LDLR* 3′UTR has been shown to associate with not only the mRNA-stabilizing factor, human antigen R (HuR), but also with decay-promoting factors, such as ZFP36 ring finger protein-like 1 and –like 2 proteins (ZFP36L1 and ZFP36L2), KH-type splicing regulatory protein (KSRP) as well as the D and I members of the heterogeneous nuclear ribonucleoprotein family (hnRNP D and hnRNP I)^16–18^. These and similar observations raise the interesting possibility that the stability of *LDLR* mRNA is determined by the sum of the positive and negative effects these *trans*-regulating factors exert on its turnover rate.

The AKT family of phospholipid-binding serine/threonine protein kinases consists of three highly homologous members, AKT1, AKT2 and AKT3, which exhibit a differential tissue-specific expression^19^. The AKT isoforms share a common structure that consists of an N-terminal pleckstrin homology (PH) domain, a central kinase domain and a C-terminal hydrophobic motif^19^. The first prerequisite for activation of AKT is interaction of its PH domain with phosphatidylinositol (3,4,5)-trisphosphate (PIP3), an event that recruits AKT to the membrane where it is activated through phosphorylation. AKT proteins regulate a wide range of cellular functions, such as cell cycle progression, apoptosis and intermediary metabolism^20^. Consequently, abnormal AKT kinase activity is implicated in the etiology of a number of diseases, such as cancer, making it an attractive target for kinase inhibitor-based drug development^20^. One such inhibitor is triciribine, a tricyclic analogue of purine which was initially identified as an inhibitor of DNA synthesis^21^. In its capacity as an inhibitor of AKT, the phosphorylated form of triciribine, triciribine phosphate, is currently in clinical testing as an anti-neoplastic agent^22,23^. Once inside the cell, triciribine is converted to triciribine phosphate by adenosine kinase^21^. Triciribine phosphate exerts it kinase inhibitory effect by binding to the PH domain of AKT, thereby preventing its recruitment to the plasma membrane^24^.

Research in our lab centers on the mechanisms that modulate the expression of LDLR. Because the regulation of LDLR by sterols is relatively well-characterized, we recently decided to focus our efforts on the investigation of other, less-characterized LDLR regulatory pathways. To this end, we chose to study the interaction between AKT and LDLR expression, because although AKT signaling has been reported to impinge on the LDLR expression machinery^25,26^, the mechanism by which it does so remains to be elucidated. During the course of our investigation into the effect of AKT inhibition of LDLR expression, we discovered that triciribine exerts a potent inducing effect on LDLR levels and decided to explore in more detail this property of triciribine. Here, we report that triciribine, while mitigating SREBP-2 transactivation activity, increases the stability of *LDLR* mRNA. The net result of these effects is a significant induction of LDLR protein levels that is accompanied by reduction in PCSK9 expression. We also show that triciribine enhances the effect of mevastatin on LDLR levels. Importantly, we found that triciribine increases the expression of LDLR in both primary human hepatocytes and mice liver.

## Results

### Stimulation of LDLR expression and function by triciribine

**Figure 1:**
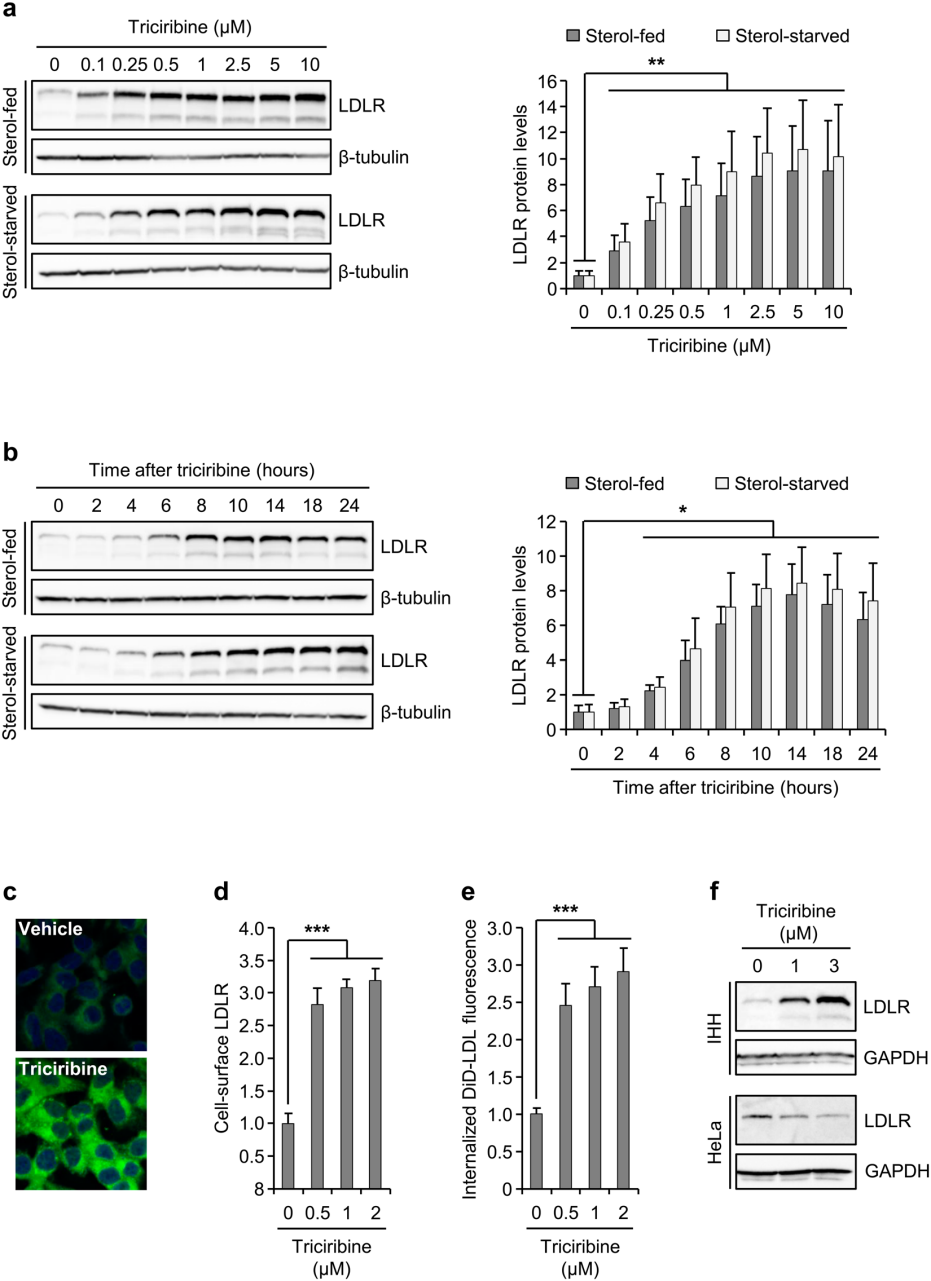
Induction of LDLR expression and activity by triciribine. (**a**) Sterol-fed or sterol-starved HepG2 cells were treated with vehicle or the indicated doses of triciribine for 14 hours and then harvested for examination by Western blotting. Left panel: One representative blot is shown (n = 5). Unprocessed blots are shown in Supplementary Fig. S7. The graph in the right panel shows quantification of the Western blots, relative to similarly cultured, vehicle-treated cells. (**b**) HepG2 cells were cultured as in a and then treated with or without 1 μM triciribine, harvested at the indicated time points and then analyzed by immunoblotting. Left panel: One representative blot is shown (n = 5). Unprocessed blots are shown in Supplementary Fig. S8. The graph in the right panel shows quantification of the Western blots, relative to similarly cultured cells harvested at 0 hour. (**c**) Sterol-fed HepG2 cells were treated with 1 µM for 14 hours and then examined by fluorescence microscopy as described in Materials and Methods. Green: LDLR, Alexa fluor 488, blue: nuclei, DAPI. One representative experiment is shown (n = 3). (**d**) Sterol-fed HepG2 cells were exposed to vehicle or 1 μM triciribine for 14 hours before analysis for mean fluorescence intensity (as a measure of cell-surface LDLR levels) by flow cytometry. The graph shows the results plotted relative to vehicle-treated control (n = 4). (**e**) Sterol-fed HepG2 cells were cultured and treated as described in d. After exposure of cells to DiD-LDL (10 μg/ml) during the last 2 hours of treatment, cells were harvested and analyzed by flow cytometry to determine the mean fluorescent intensity of internalized DiD-LDL. The graph shows the results plotted relative to vehicle-treated control (n = 4). (**f**) Sterol-fed immortalized human hepatocytes (IHH) and HeLa cells were treated with vehicle or the indicated concentrations of triciribine for 14 hours and then harvested and analyzed by Western blotting. One representative blot is shown (n = 3). Unprocessed blots are shown in Supplementary Fig. S9. Error bars represent SD. *p < 0.05, **p < 0.01 and ***p < 0.001 compared with matched vehicle-treated cells (a, d and e) or matched cells harvested at 0 hour (b).

To examine the effect of AKT inhibition on LDLR expression, we chose triciribine, a tricyclic nucleoside that inhibits all AKT isoforms with a high degree of selectivity^22^. Using HepG2 cells, a human hepatoma cell line, we assessed whether triciribine influences LDLR protein levels and if the effect is influenced by the presence or absence of sterols in the culture medium. To this end, HepG2 cells were first cultured in medium containing fetal bovine serum (FBS) or lipoprotein-deficient serum (LPDS; henceforth, sterol-fed and sterol-starved, respectively). Sterol-starved cells have lower cholesterol content than sterol-fed cells^27,28^. Cells were then treated with different concentrations of triciribine for 14 hours and examined for LDLR expression by Western blotting. Figure 1a shows that triciribine elevated the levels of LDLR in both sterol-fed and sterol-starved cells in a dose-responsive manner up to a concentration of 5 µM. The sub-maximal concentration of 1 µM was used for the remainder of this study unless otherwise indicated. To examine the induction kinetics of LDLR in response to triciribine, sterol-fed and sterol-starved HepG2 cells were treated with 1 µM triciribine and harvested at different times up to 24 hours for analysis of LDLR levels by immunoblotting. LDLR was significantly induced within 4 hours after triciribine treatment and continued to increase until 14 hours after which triciribine exhibited a slight loss of its LDLR-inducing effect (Fig. 1b). Importantly, examination of the phosphorylation status of AKT on S473, which serves as an indicator of its kinase activity, showed that the onset of LDLR induction by triciribine was preceded by a robust inhibition of AKT activity (Supplementary Fig. S1). The immunoblotting results for LDLR were further confirmed by the results obtained with fluorescence microscopy showing that cells exposed to triciribine expressed higher amount of LDLR protein (Fig. 1c). Thus, triciribine increases the expression of LDLR under both basal and sterol-deficient culture conditions. To assess the impact of triciribine on the function of LDLR, we analyzed the effect of triciribine on cell-surface LDLR expression. Fig. 1d shows that treatment with triciribine potently increased cell-surface LDLR levels. Importantly, the induction of LDLR surface expression by triciribine correlated with enhanced internalization of DiD-LDL (Fig. 1e). These results show that the induction of LDLR by triciribine translates to an increase in LDLR function.

Finally, to ascertain that the observed effect is not specific to HepG2 cells, we examined the effect of triciribine on LDLR expression in hTERT-immortalized human hepatocytes hepatocytes (IHH) and the non-hepatic cell line, HeLa. Similar to HepG2, IHH cells responded to triciribine treatment with an increased expression of LDLR (Fig. 1f). In contrast, triciribine failed to increase LDLR levels in HeLa cells. Taken together, these results suggest that the LDLR-inducing effect of triciribine may be restricted to cells of hepatic origin.

### Triciribine represses the turnover of *LDLR* mRNA

**Figure 2:**
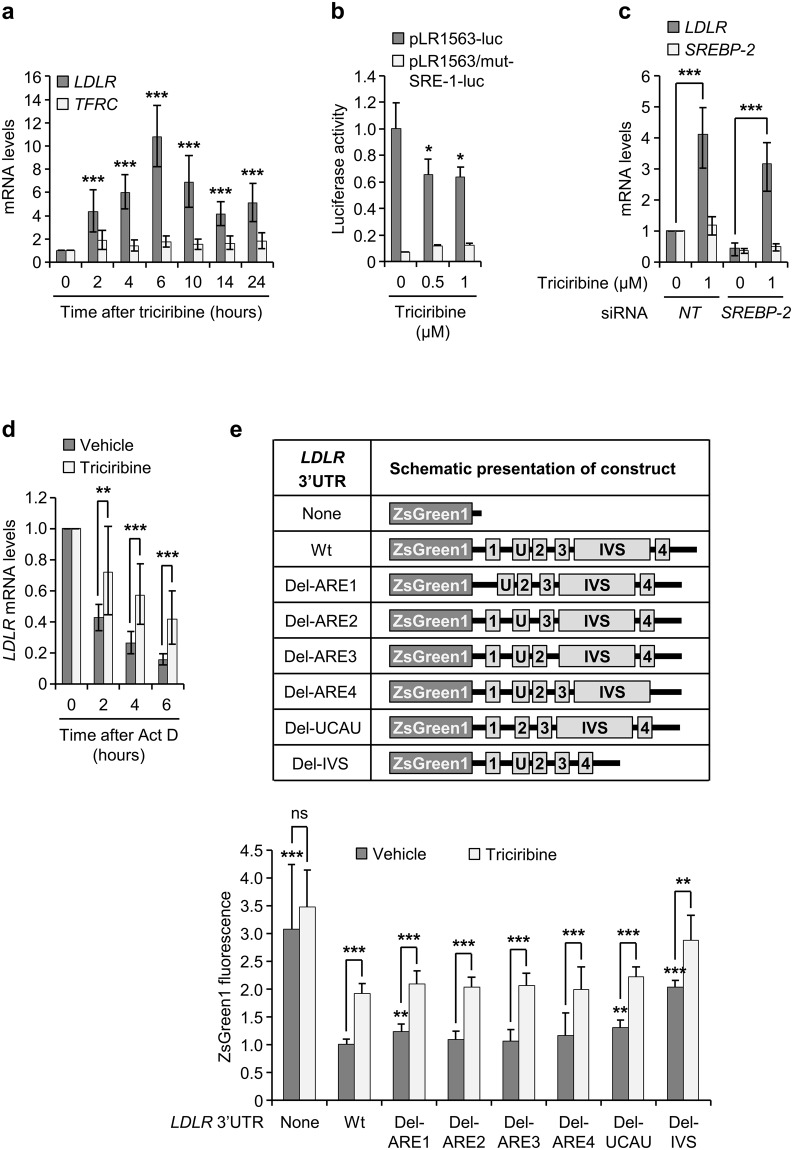
Triciribine induces *LDLR* mRNA levels in the absence of SREBP-2 activity. (**a**) Sterol-fed HepG2 cells were treated with or without 1 μM triciribine before harvesting at the indicated times. Cells were then processed for isolation of mRNA and determination of *LDLR*, *TFRC* and *GAPDH* mRNA levels by qPCR assay. The plot shows *LDLR* and *TFRC* mRNA levels plotted relative to cells harvested at time 0 (n = 4). (**b**) Sterol-fed HepG2 cells were transfected with firefly luciferase reporter constructs containing either wt (pLR1563-luc) or mutant (pLR1563/mutSRE-1-luc) *LDLR* promoter and Renilla luciferase reporter. At 16 hours after transfection, cells were exposed to the indicated concentrations of triciribine for 14 hours and then harvested for determination of *LDLR* promoter activity. The graph shows the results plotted relative to vehicle-treated cells that were transfected with pLR1563-luc (n = 4). (**c**) Sterol-fed HepG2 cells were transfected with a non-targeting siRNA (*NT* siRNA) or an *SREBP-2*-specific siRNA. Twenty four hours after transfection, vehicle or triciribine was added to the cultures and cells were incubated for another 14 hours before harvesting for qPCR analysis of *LDLR*, *SREBP-2* and *GAPDH* mRNA levels. The levels of *LDLR* and *SREBP-2* mRNAs were then plotted relative to vehicle-treated, *NT* siRNA-transfected cells (n = 4). (**d**) At 14 hours after treatment of sterol-fed HepG2 cells with vehicle or 1 µM triciribine, actinomycin D (Act D; 5 µg/ml) was added to the cultures and then harvested at the indicated time points for qPCR analysis of *LDLR* and *GAPDH* mRNA levels. The plot shows *LDLR* mRNA levels relative to respective vehicle-treated controls (n = 4). (**e**) Upper panel: schematic presentation of the chimeric ZsGreen1-LDLR-3′UTR constructs. Lower panel: at 24 hours after transfection of sterol-fed HepG2 cells with one of the constructs, cells were treated with 1 µM triciribine and then harvested for determination of mean ZsGreen1 fluorescence intensity (as an indicator of *ZsGreen1* transcript levels) by flow cytometry. The graph shows the results plotted relative to vehicle-treated cells that were transfected with ZsGreen1-LDLR-3′UTR-wt construct (n = 4). AREs are represented with numerically labelled rectangles. Letter U refers to UCAU repeats. IVS, intervening sequence. mRNA data are displayed with error bars representing the 95% confidence interval. Otherwise, error bars represent SD. *p < 0.05, **p < 0.01 and ***p < 0.001 compared with matched cells harvested at time 0 (a and d), vehicle-treated cells that were transfected with pLR1563-luc (**b**), matched vehicle-treated cells that were transfected with *NT* siRNA (**c**) or matched vehicle-treated cells (**e**).

As a first step towards understanding the mechanism that underlies the triciribine-mediated induction of LDLR, we examined the effect of triciribine on the expression of *LDLR* mRNA. As shown in Fig. 2a, *LDLR* mRNA was rapidly induced by triciribine in sterol-fed HepG2 cells, and reached a maximum level at about 6 hours after which it gradually declined to approximately half its peak level by the end of the experiment. Because LDLR expression is mainly regulated at the level of transcription, we wished to assess whether triciribine induces *LDLR* gene transcription. To this end, we first transfected sterol-fed HepG2 cells with pLR1563-luc vector, a construct that encodes the firefly luciferase gene under the control of the human *LDLR* promoter region +58 to −1563^29^, and examined them for luciferase activity after exposure to triciribine. Surprisingly, treatment of cells with triciribine led to reduction, rather than induction, of luciferase activity (Fig. 2b), suggesting that activation of *LDLR* gene expression does not account for triciribine-mediated induction of *LDLR* mRNA levels. To determine whether this suggestion holds true in the endogenous cellular setting, we examined the effect of triciribine on *LDLR* mRNA levels in cells whose SREBP-2 was knocked down by siRNA. If induction of *LDLR* gene is responsible for triciribine-mediated upregulation of *LDLR* mRNA, then inhibition of SREBP-2, which confers regulability on *LDLR* gene expression^6^, should abrogate the inducing effect of triciribine on *LDLR* mRNA levels. As shown in Fig. 2c, triciribine retained its ability to induce *LDLR* mRNA levels in the absence of SREBP-2. These results show that triciribine utilizes a post-transcriptional mechanism to increase *LDLR* mRNA levels.

Stabilization of mRNA provides a plausible explanation for the triciribine-mediated accumulation of *LDLR* mRNA in the absence of *LDLR* gene induction. To examine the validity of this notion, we assessed the effect of triciribine on *LDLR* mRNA stability using actinomycin D (Act D). As shown in Fig. 2d, triciribine significantly increased the stability of *LDLR* mRNA. Estimation of *LDLR* mRNA half-life showed that triciribine increased the half-life of *LDLR* mRNA from about 1.9 hours to approximately 4.8 hours. Thus, triciribine induces the accumulation of *LDLR* mRNA through induction of its stability.

Mammalian cells utilize a number of different mRNA decay pathways to regulate the stability of mRNA^30^. One such mechanism relies on the *cis*-regulatory elements that reside in the 3′UTR of mRNA. The *LDLR* 2.5 kb long 3′UTR harbors four AU-rich elements (AREs) and a region encompassing four clustered UCAU repeats that have been shown to impact the turnover rate of *LDLR* mRNA^8,11,13^. In addition, close inspection of *LDLR* 3′UTR sequence showed the region between ARE3 and ARE4, referred to herein as intervening sequence (IVS), to be disproportionally AU-rich. To determine whether these sequences in the 3′UTR of *LDLR* mRNA play a role in the stabilizing effect of triciribine on *LDLR* mRNA, we cloned wt as well as different deletion mutants of *LDLR* 3′UTR into the pmR-ZsGreen1 reporter plasmid. These constructs express the fluorescent ZsGreen1 protein from a *ZsGreen1-LDLR-3′UTR* chimeric mRNA. Translation of these transcripts produces ZsGreen1 proteins whose fluorescence serves as an indicator for the stability of the chimeric transcript. As expected, inclusion of wt *LDLR* 3′UTR in pmR-ZsGreen1 plasmid potently reduced the amount of ZsGreen1 fluorescence, confirming the destabilizing effect of *LDLR* 3′UTR in this experimental system (Fig. 2e). Importantly, cells that were transfected with pmR-ZsGreen1-LDLR-3′UTR-wt exhibited a one-fold increase in fluorescence when treated with triciribine. However, neither ARE1, ARE2, ARE3, ARE4 nor UCAU deletions had an appreciable impact on the detected fluorescence signal in triciribine-treated cells. In contrast, deletion of IVS in *LDLR* 3′UTR not only augmented ZsGreen1 levels in untreated cells, but also partially alleviated (30%) the effect of triciribine on the expression of ZsGreen1 fluorescence. To exclude the possibility that the effects observed with pmR-ZsGreen1-LDLR-3′UTR-Del-IVS result from shortening of *LDLR* 3′UTR, we replaced the IVS region (1202 bases) in pmR-ZsGreen1-LDLR-3′UTR-wt with a 1155 bases long portion of the transferrin receptor (*TFRC*) coding region to generate pmR-ZsGreen1-LDLR-3′UTR-IVS>TFRC plasmid. This vector expresses a *ZsGreen1-LDLR-3′UTR* chimeric mRNA that is of similar size to *ZsGreen1-LDLR-3*′*UTR-wt* but lacks the IVS region. As shown in Supplementary Fig. S2, the level of fluorescence expression generated by pmR-ZsGreen1-LDLR-3′UTR-IVS>TFRC was similar to that of pmR-ZsGreen1-LDLR-3′UTR-Del-IVS. Furthermore, replacement of IVS with the TFRC coding sequence had an inhibitory effect (25%) on triciribine-mediated induction of fluorescence expression that was similar to that of IVS deletion. Taken together, these results suggest that triciribine exerts its *LDLR* mRNA-stabilizing effect in part through one or several destabilizing regulatory elements that are located between ARE3 and ARE4.

### Triciribine inhibits PCSK9 expression

**Figure 3:**
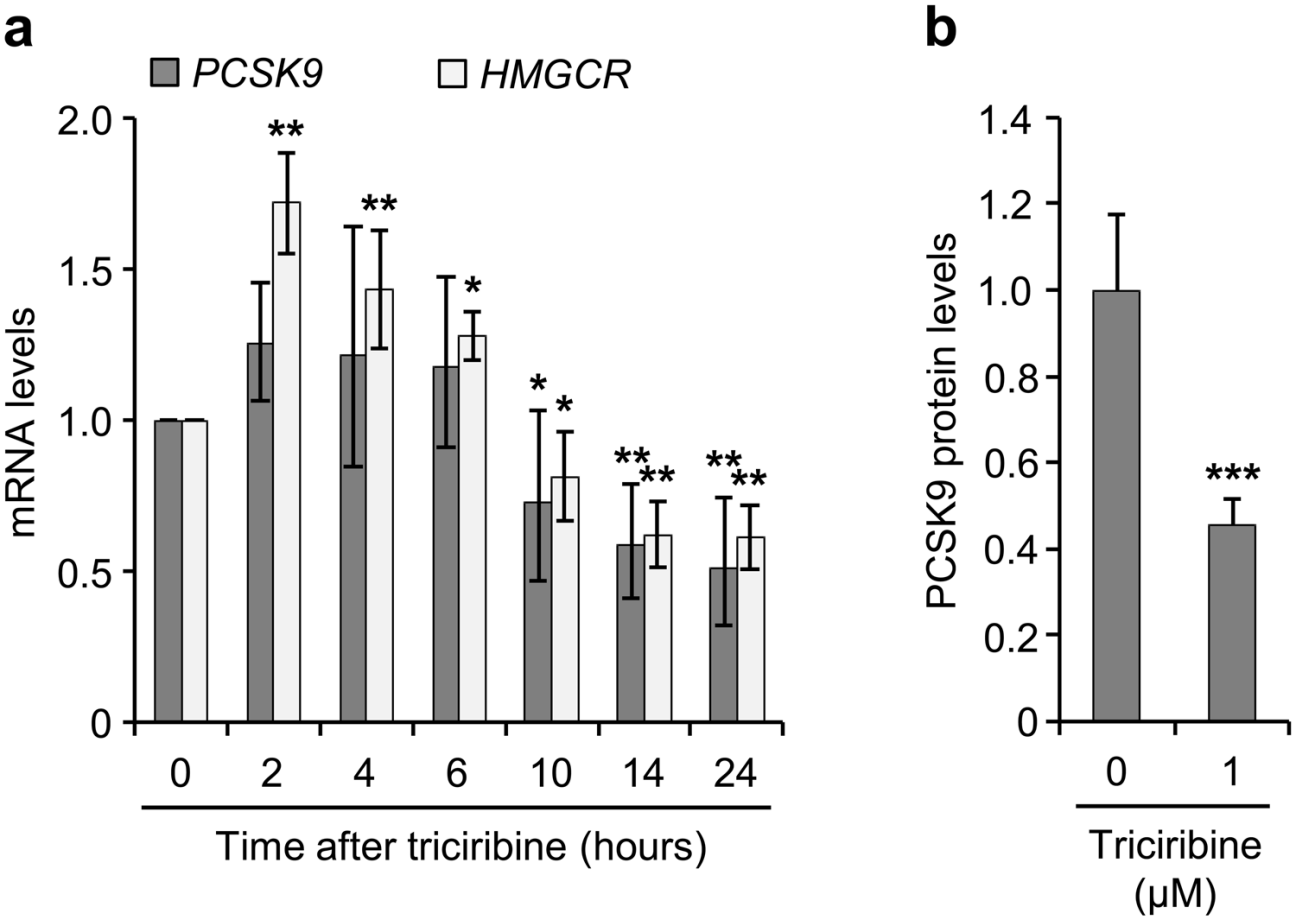
Inhibition of PCSK9 by triciribine. (**a**) Sterol-fed HepG2 cells were treated with or without 1 μM triciribine before harvesting at the indicated times. Cells were then processed for isolation of mRNA and determination of *PCSK9*, *HMGCR* and *GAPDH* mRNA levels by qPCR. The levels of *PCSK9* and *HMGCR* mRNAs were then plotted relative to cells harvested at 0 hour (n = 4). (**b**) Sterol-fed HepG2 cells were treated with or without 1 µM triciribine. The media were recovered at 14 hours post-treatment and subjected to analysis for PCSK9 levels as described in Materials and Methods. The graph shows the results plotted relative to vehicle-treated control (n = 4). Error bars represent the 95% confidence interval (**a**) or SD (**b**). *p < 0.05, **p < 0.01 and ***p < 0.001 when compared with matched cells harvested at 0 hour (**a**) or vehicle-treated cells (**b**).

PCSK9 plays an important role in cholesterol homeostasis as a result of its ability to reduce cell-surface LDLR levels^7^. The promoter of *PCSK9* gene contains a serum response element (SRE) that makes its transcription subject to regulation by SREBP-2^31^. Given the above observation that triciribine inhibited the SREBP-2-dependent transcription, we set out to investigate the effect of triciribine on *PCSK9* mRNA levels. Fig. 3a shows that triciribine reduced the levels of *PCSK9* mRNA. Similarly, expression of *HMGCR* mRNA whose gene is also subject to regulation by SREBP-2^32,33^ was inhibited by triciribine (Fig. 3a). These results suggest that inhibition of SREBP-2-regulated transcription is a general feature of triciribine. PCSK9 exerts its inhibitory effect on LDLR following its secretion into the extracellular milieu. Therefore, we wished to examine whether the triciribine-mediated inhibition of PCSK9 expression is associated with a decrease in the level of secreted PCSK9. To this end, we carried out ELISA measurements of PCSK9 secreted into the media of HepG2 cells that were cultured in the absence or presence of triciribine and found that PCSK9 secretion from cells that were exposed to triciribine was significantly attenuated (Fig. 3b).

### Triciribine augments the mevastatin-mediated upregulation of LDLR levels and LDL uptake

**Figure 4:**
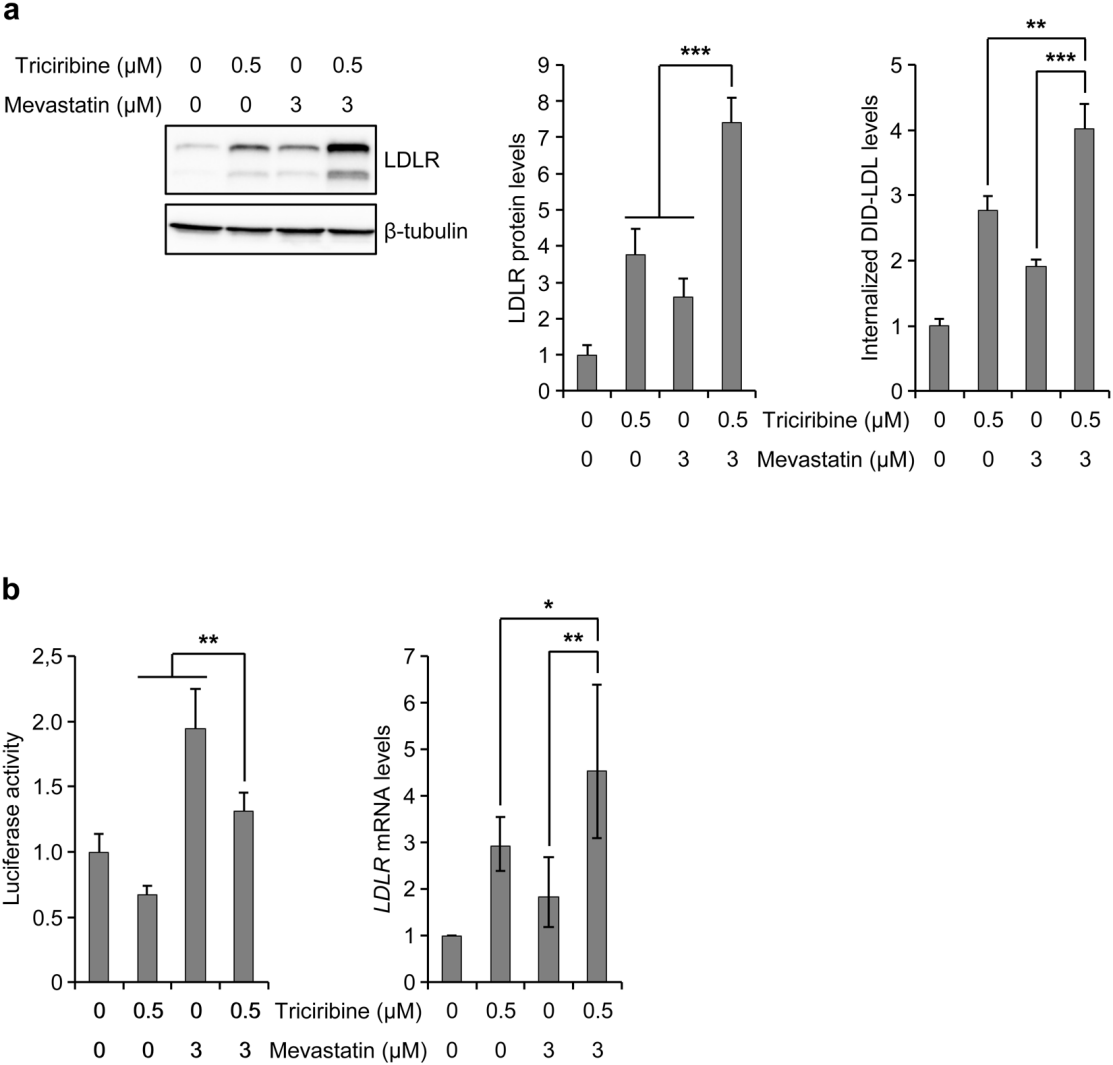
Triciribine enhances the effect of mevastatin on LDLR and LDL uptake. (**a**) Sterol-starved HepG2 cells were treated with vehicle or mevastatin for 24 hours before exposure to triciribine. At 10 hours after addition of triciribine, DiD-LDL (10 μg/ml) was added to the cultures and incubated for another 2 hours before harvest (n = 4). Each sample was divided into two halves, of which one was analyzed by Western blotting. Left panel shows one representative blot. Unprocessed blots are shown in Supplementary Fig. S10. The graph in the middle panel shows quantification of the Western blots, relative to vehicle-treated cells. Right panel: the other halves of samples were analyzed by flow cytometry to determine the mean fluorescence intensity of internalized DiD-LDL. Results were then plotted relative to vehicle-treated control. (**b**) Sterol-fed HepG2 cells were co-transfected with pLR1563-luc and Renilla luciferase reporter. At 16 hours after transfection, cells were washed twice and then cultured under sterol-starved conditions in the absence or presence of mevastatin for 24 hours before treatment with triciribine for a further 12 hours before harvest (n = 4). Each sample was divided into two halves, of which one was analyzed for determination of *LDLR* promoter activity. The graph in the left panel shows the results plotted relative to vehicle-treated cells. The other halves of samples were subjected to qPCR for quantification of *LDLR* and *GAPDH* mRNA levels. The graph in the right panel shows *LDLR* mRNA levels relative to vehicle-treated cells. mRNA data are displayed with error bars representing the 95% confidence interval. Otherwise, error bars represent SD. *p < 0.05, **p < 0.01 and ***p < 0.001.

The ability of triciribine to increase the *LDLR* mRNA pool size through mRNA stabilization suggested that in a combinatorial treatment, it would potentiate the LDLR-inducing effect of statins. To examine this assumption, we treated sterol-starved HepG2 cells with triciribine and mevastatin, either alone or in combination, and analyzed them for LDLR levels and LDL uptake. As shown in Fig. 4a, treatment of cells with both triciribine and mevastatin enhanced the mevastatin-mediated induction of LDLR protein. Similarly, examination of cellular uptake of DiD-LDL showed that triciribine significantly enhanced the effect of mevastatin on LDL internalization.

Stimulation of mevastatin-mediated induction of LDLR by triciribine suggests that triciribine ameliorates the mevastatin-induced activation of SREBP-2, an implication that contrasts with the result that triciribine exerts an inhibitory effect on SREBP-2 activity (Fig. 2b and Supplementary Fig. S3). To address this contradiction, we set out to examine the *LDLR* promoter activity and mRNA levels in cells that were co-treated with triciribine and mevastatin. To this end, we treated HepG2 cells that were transfected with pLR1563-luc vector with triciribine and mevastatin, either alone or in combination, and examined them for luciferase activity and *LDLR* mRNA levels. As expected, luciferase activity was partially inhibited in cells that were treated with triciribine, whereas it was induced in mevastatin-treated cells (Fig. 4b). Importantly, although cells that were co-treated with triciribine and mevastatin contained somewhat lower levels of luciferase activity than cells that were treated with mevastatin alone, they expressed higher levels of *LDLR* mRNA, apparently as a consequence of the *LDLR* mRNA-stabilizing effect of triciribine. This result indicates that the *LDLR* mRNA-stabilizing effect of triciribine not only compensates for its hampering effect on SREBP-2 activity, but also contributes to its stimulating effect on mevastatin-mediated induction of *LDLR* mRNA.

### Induction of LDLR by triciribine requires ERK activity

**Figure 5:**
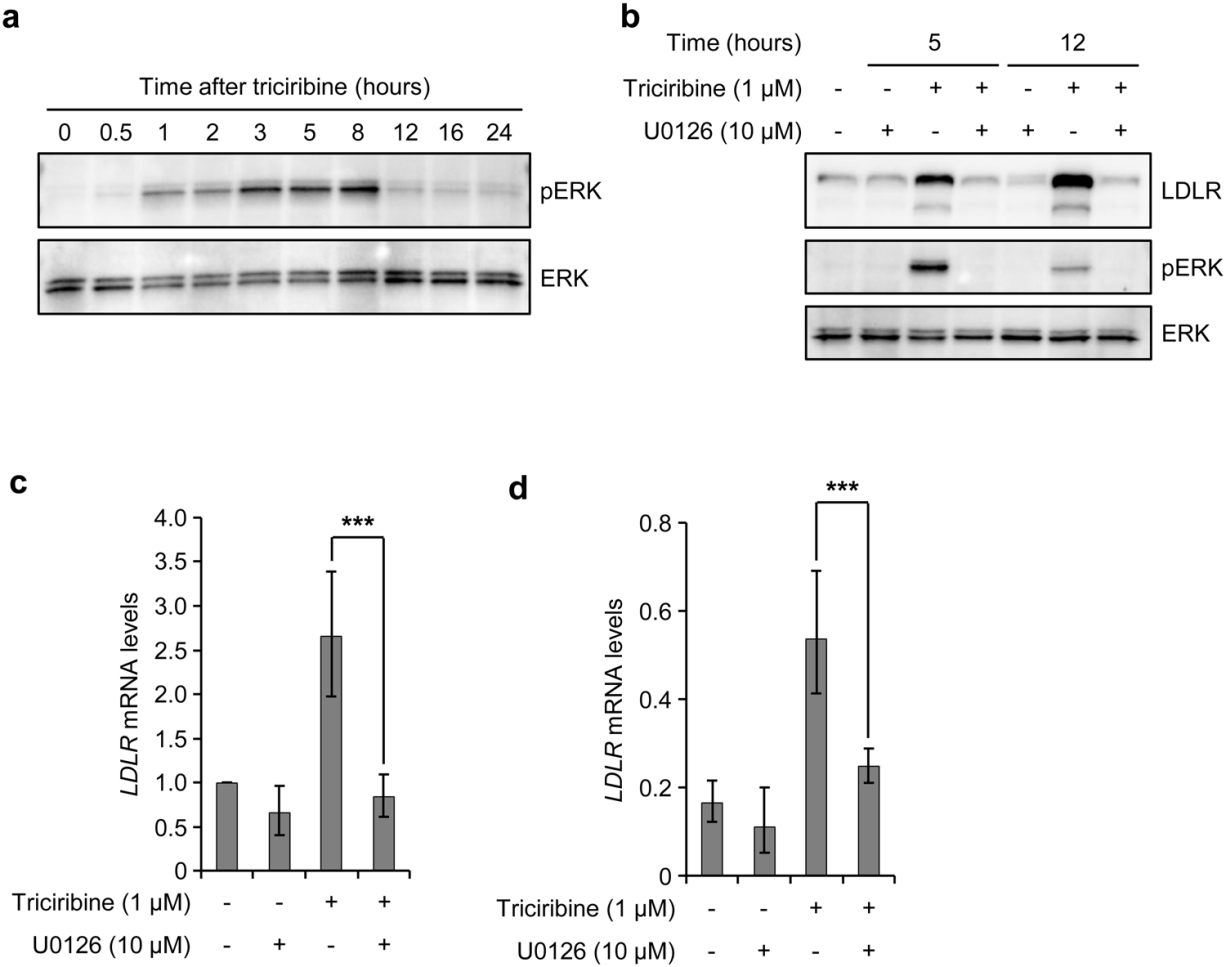
Stabilization of *LDLR* mRNA by triciribine is dependent on ERK activity. (**a**) Sterol-fed HepG2 cells were treated with vehicle or 1 µM triciribine and harvested at the time points indicated for Western blot analysis. One representative blot from four independent experiments is shown. Unprocessed blots are shown in Supplementary Fig. S11. (**b**) sterol-fed HepG2 cells were pretreated with U0126 for 1 hour before exposure to triciribine. Cells were harvested at the indicated time points after addition of triciribine and analyzed by Western blotting. One representative blot is shown (n = 4). Unprocessed blots are shown in Supplementary Fig. S12. (**c**) Sterol-fed HepG2 cells were treated as in b, harvested 14 hours after addition of triciribine and then processed for isolation of mRNA and determination of *LDLR* and *GAPDH* mRNA levels by qPCR assay. The plot shows *LDLR* mRNA levels relative to vehicle-treated cells (n = 4). (**d**) Sterol-fed HepG2 cells were treated as in b. At 14 hours after addition of triciribine, cells were treated with actinomycin D (Act D; 5 µg/ml) and then harvested after 6 hours for qPCR analysis of *LDLR* and *GAPDH* mRNA levels. The plot shows *LDLR* mRNA levels relative to respective vehicle-treated controls that were harvested before addition of Act D (0 hour), the values of which was set at 1 (n = 4). Error bars represent the 95% confidence interval. ***p < 0.001.

The effects of triciribine on *LDLR* mRNA stability and PCSK9 levels were reminiscent of those elicited by berberine and 5-Azacytidine (5-AzaC)^11,34,35^. This similarity suggested that triciribine might use a mechanism analogous to that of berberine or 5-AzaC to increase the stability of *LDLR* mRNA. We therefore proceeded to examine whether ERK was involved in the stabilizing effect of triciribine on *LDLR* mRNA. We first assessed the effect of triciribine on phosphorylation of ERK as an indicator of its kinase activity. Treatment of cells with triciribine led to a rapid induction of ERK phosphorylation (within 1 hour) which reached its peak value at 3 hours after which it decreased to levels slightly above basal values (Fig. 5a). Of note is the observation that induction of *LDLR* mRNA is preceded by the kinetics of ERK phosphorylation (see Fig. 1b). To examine whether ERK plays a role in the inducing effect of triciribine on LDLR levels, we treated the cells with the MEK/ERK inhibitor, U0126, in the absence or presence of triciribine for 5 or 12 hours and then examined them for ERK phosphorylation and LDLR protein levels by Western blotting. U0126 was found to abrogate the phosphorylation of ERK both in the absence or presence of triciribine, an event that correlated with inhibition of triciribine-mediated induction of LDLR protein and *LDLR* mRNA (Fig. 5b,c). Finally, to ascertain the nature of this inhibitory effect of U0126, we examined the effect of ERK inhibition on the stabilizing effect of triciribine on *LDLR* mRNA. As shown in Fig. 5d, U0126 significantly mitigated the triciribine-induced stabilization of *LDLR* mRNA. Taken together, these results show that triciribine stabilizes *LDLR* mRNA in an ERK activity-dependent manner.

### Triciribine phosphate mimics the effect of triciribine on LDLR

**Figure 6:**
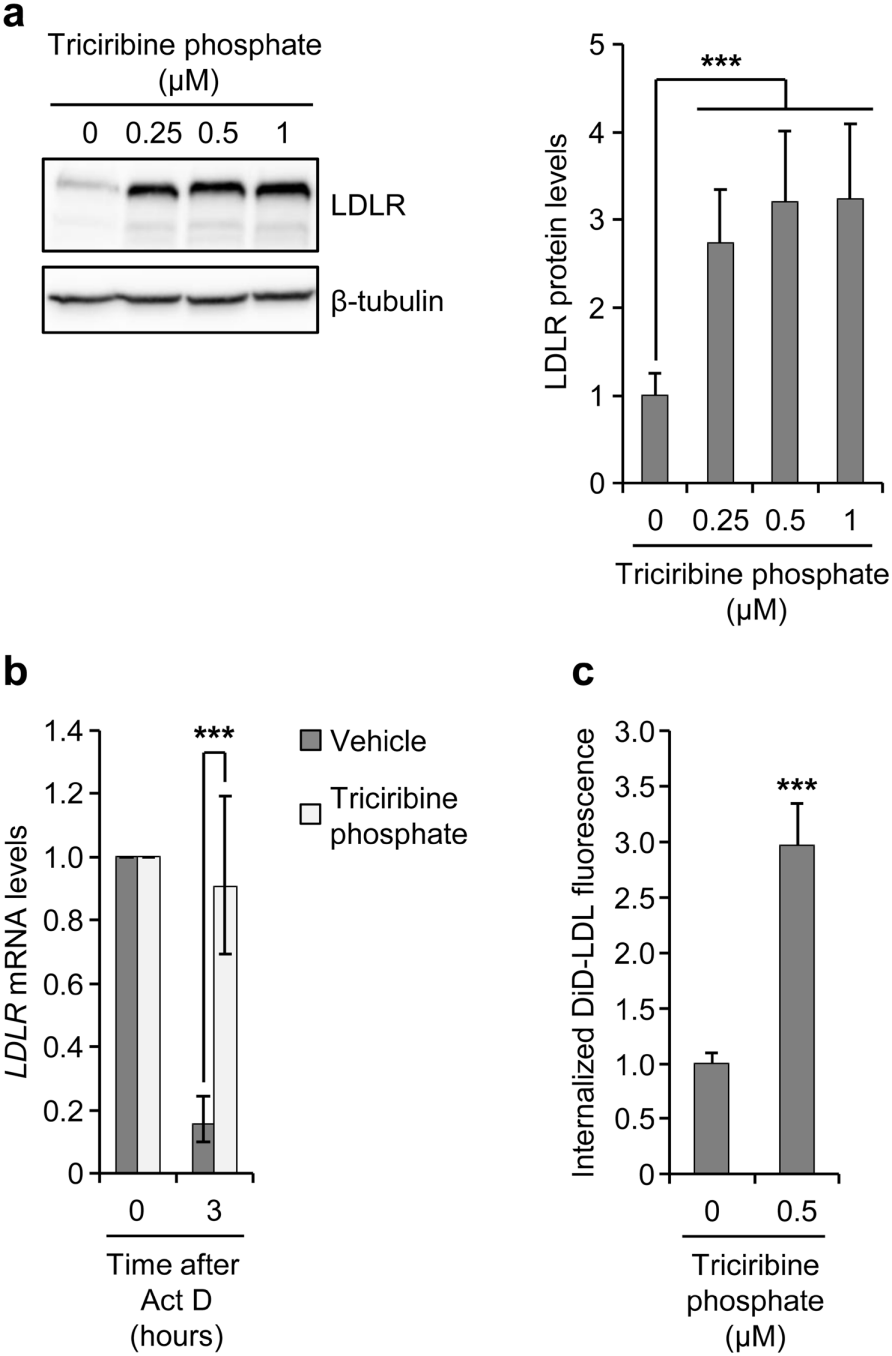
Triciribine phosphate mimics the effect of triciribine on LDLR expression and function. (**a**) Sterol-fed HepG2 cells were treated with vehicle or the indicated doses of triciribine phosphate. At 14 hours after incubation, cells were harvested and subjected to Western blot analysis. The left panel shows one representative blot from four independent experiments. Unprocessed blots are shown in Supplementary Fig. S13. The graph in the right panel shows quantification of the Western blots, relative to vehicle-treated cells. (**b**) Sterol-fed HepG2 cells were treated with vehicle or 0.5 µM triciribine phosphate for 14 hours before addition of actinomycin D (Act D; 5 μg/ml). At the indicated time points after addition of Act D, cells were harvested and analyzed by qPCR for quantification of *LDLR* and *GAPDH* mRNA levels. The plot shows *LDLR* mRNA levels relative to cells at time 0 (n = 4). (**c**) Sterol-fed HepG2 cells were treated with vehicle or triciribine phosphate for 14 hours. After exposure of cells to DiD-LDL (10 μg/ml) during the last 2 hours of treatment, cells were harvested and analyzed by flow cytometry to determine the mean fluorescence intensity of internalized DiD-LDL. The graph shows the results plotted relative to vehicle-treated control (n = 4). Error bars represent SD (a and c) or the 95% confidence interval (**b**) ***p < 0.001 when compared with vehicle-treated cells (**a**,**c**) or matched, vehicle-treated cells harvested at 0 hour (**b**).

Once inside the cell, triciribine is converted to its active phosphorylated derivative, triciribine phosphate that is currently in phase II human clinical trials^21,36–38^. Consequently, we considered it important to examine whether the effect of triciribine phosphate on LDLR levels and LDL uptake were similar to those produced by triciribine. For this purpose, we treated HepG2 cells with different concentrations of triciribine phosphate and then examined them for the expression of LDLR protein. As shown in Fig. 6a, treatment of cells with triciribine phosphate strongly upregulated LDLR protein levels. Next, we extended our analysis to the effect of triciribine phosphate on *LDLR* mRNA stability and found that triciribine phosphate exerted a potent stabilizing effect on *LDLR* mRNA (Fig. 6b). Finally, to assess the effect of triciribine phosphate on LDLR functionality, we analyzed internalization of DiD-LDL following exposure of cells to triciribine phosphate. As shown in Fig. 6c, treatment of cells with triciribine phosphate led to a significant induction of cellular DiD-LDL uptake. In sum, these results demonstrate that triciribine phosphate affects LDLR expression and LDL uptake in a fashion similar to that of triciribine.

### Triciribine stimulates LDLR expression in primary human hepatocytes and mouse liver

**Figure 7:**
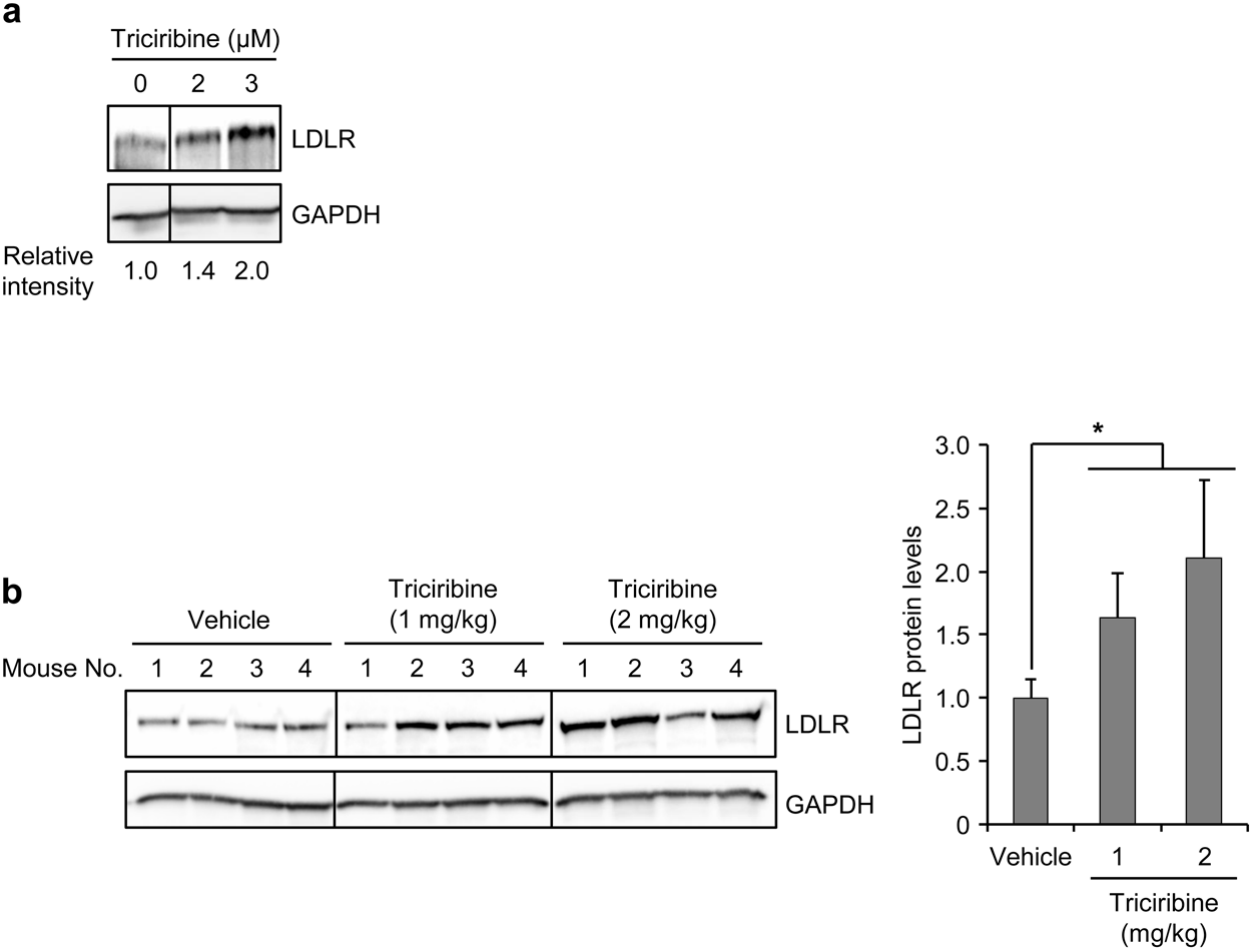
Effect of triciribine on LDLR expression in primary human hepatocytes and mouse liver. (**a**) Primary human hepatocytes cultured in Maintenance Medium were treated with vehicle or the indicated concentrations of triciribine. After 14 hours of treatment, cells were harvested and analyzed by Western blotting. After quantification of the blot by densitometry, the relative level of LDLR signal intensity was determined by normalizing LDLR band intensity to GAPDH band intensity. One representative experiment of two is shown. Vertical lines denote repositioning of lanes run on the same gel. Unprocessed blots are shown in Supplementary Fig. S14. (**b**) C57BL/6 J mice fed on standard chow were treated daily with vehicle or the indicated doses of triciribine for two weeks (each group consisted of four mice). Three hours after the last treatment, the animals were sacrificed to harvest the liver. Lysates prepared from liver samples were analyzed by immunoblotting with the indicated antibodies. Vertical lines denote repositioning of lanes run on the same gel. Unprocessed blots are shown in Supplementary Fig. S15. The immunoblot in the left panel was scanned and the LDLR band intensities were normalized to those of GAPDH. The mean values for triciribine-treated samples were then plotted relative to the value from vehicle-treated samples. Error bars represent SD. *p < 0.05 when compared with vehicle control.

To extend our findings obtained in cell lines to primary cells, we exposed cultured primary adult human hepatocytes to different concentrations of triciribine and analyzed them for LDLR protein levels by immunoblotting. Triciribine induced LDLR levels in primary human hepatocytes, albeit at concentrations higher than those that were needed to exert a similar effect in cell lines (Fig. 7a). Next, we sought to obtain an indication of whether triciribine exerts an LDLR-inducing effect in an *in vivo* setting. To this end, we first treated C57BL/6 J mice with two different daily doses of triciribine (1 mg/kg or 2 mg/kg) for two weeks and then analyzed them for hepatic LDLR levels. Fig. 7b shows that treatment of mice with either doses of triciribine for two weeks significantly increased the levels of hepatic LDLR. Interestingly, triciribine, similar to its effect on ERK phosphorylation in HepG2 cells, induced the phosphorylation of hepatic ERK (Supplementary Fig. S4). Taken together, these results show that triciribine exerts an LDLR-inducing effect in both primary human hepatocytes and mouse liver.

## Discussion

As a result of its ability to internalize cholesterol into the cells, LDLR plays a crucial role in modulation of circulating levels of cholesterol^4,5^. It is therefore unsurprising that drugs that increase LDLR levels by either inducing its expression or inhibiting its PCSK9-mediated degradation have proven to be the most effective treatments for hypercholesterolemia^39–41^. Here, we report identification of a new agent, triciribine, as an effective LDLR-inducing agent that not only induces the expression of LDLR but also reduces PCSK9 levels.

Using *in vitro* cultures of the human hepatoma cell line HepG2 as a model system to study modulation of LDLR expression, we found that triciribine, a highly specific inhibitor of AKT kinases, induces the expression of LDLR through augmentation of its mRNA stability. Examination of ZsGreen1 fluorescence in cells that expressed chimeric *ZsGreen1* mRNAs containing either wt or deletion mutants of *LDLR* 3′UTR revealed that none of the four *LDLR* mRNA AREs or the cluster of UCAU repeats alone is utilized by triciribine to exert its mRNA-stabilizing effect. Although this result suggests that these *cis*-regulatory elements are not targeted by triciribine in isolation, it does not preclude the possibility that these elements mediate the stabilizing effect of triciribine on *LDLR* mRNA in a combinatorial manner. Indeed, evidence suggests that *cis*-regulatory elements in 3′UTR often act in a combinatorial and perhaps redundant manner to control mRNA stability^42–44^. For instance, destabilization of *IL-17* mRNA by tristetrapolin has been shown to depend on a cluster of several ARE motifs within the *IL-17* 3′UTR^45^. Similarly, it has been reported that p38α targets 3 distinct AREs in *IL-6* 3′UTR to stabilize *IL-6* mRNA^46^. Therefore, it is reasonable to suggest that triciribine might depend on the concomitant action of several *cis*-acting elements in *LDLR* 3′UTR for its mRNA-stabilizing effect. Interestingly, examination of ZsGreen1 fluorescence in cells that expressed the chimeric *ZsGreen1-LDLR-3*′*UTR-Del-IVS* or *ZsGreen1-LDLR-3*′*UTR-IVS>TFRC* revealed that the intervening region between ARE3 and ARE4 plays an partial role in both basal *LDLR* mRNA turnover, as well as the mRNA-stabilizing effect of triciribine. *LDLR* 3′UTR has been shown to be subject to m^6^A modification, an event that facilitates its binding to the YTH-domain family member 2 (YTHDF2), thus resulting in *LDLR* mRNA decay^47^. This report, combined with our observation that the IVS region contains several sites that closely match the consensus signal for m^6^A methylation^48^, raise the possibility that triciribine may inhibit methylation of *LDLR* mRNA in the IVS region to protect it from binding to the mRNA-destabilizing m^6^A readers. If so, it will be interesting to investigate whether triciribine inhibits m^6^A writers or stimulates m^6^A erasers to abrogate methylation of *LDLR* mRNA.

The finding that triciribine reduces *LDLR* promoter activity suggests that it exerts an inhibitory effect on SREBP-2 activity. This idea is corroborated by the inhibitory effect of triciribine on *HMGCR* and *PCSK9* mRNA levels, whose genes are also regulated by SREBP-2. Because these results were obtained with cells that were cultured in the presence of FBS, it may be suggested that through induction of cellular uptake of cholesterol, triciribine increases intracellular cholesterol levels, an event that is expected to trigger the feedback inhibition of SREBP-2. However, triciribine was found to also decrease *HMGCR* and *PCSK9* mRNA levels in cells that were cultured in the presence of LPDS indicating that inhibition of SREBP-2 activity occurs as a direct response to triciribine. This notion is substantiated by the finding that triciribine inhibits the expression of the C-terminal fragment of cleaved SREBP-2 (Supplementary Fig. S3).

Interestingly, triciribine shares a number of similar traits with berberine and 5-AzaC: 1) inhibition of DNA synthesis, 2) induction of ERK activity, 3) stabilization of *LDLR* mRNA and 4) inhibition of PCSK9 expression^11,34,35,49–51^. These similarities raise the possibility that triciribine exerts the effects that are presented in this report in its capacity as an inhibitor of DNA synthesis. Although we cannot formally disprove this suggestion, we are inclined to believe that triciribine-mediated induction of LDLR expression occurs as a result of its ability to inhibit AKT activity. This is primarily because GSK690693, another selective AKT inhibitor, also increases the expression of LDLR (Supplementary Fig. S5). Furthermore, triciribine exhibits a number of functional dissimilarities with berberine and 5-AzaC. For instance, while berberine has no effect on proteolytic activation of SREBP-2^11^, triciribine appears to inhibit cleavage of SREBP-2 (Supplementary Fig. S3). Similarly, induction of *LDLR* mRNA by 5-AzaC has been shown to be associated with increase in IRE1α levels^34^, whereas triciribine inhibits the expression of IRE1α (Supplementary Fig. S6).

Berberine is reported to exert its *LDLR* mRNA-stabilizing effect by modulating the activity of mRNA-destabilizing factors hnRNP I and KSRP^18^. Therefore, we believe that future experiments designed to identify *trans*-regulatory proteins that mediate the stabilizing effect of triciribine on *LDLR* mRNA should consider hnRNP I and KSRP as plausible targets of triciribine. Equally important is assessment of the possible role of ZFP36L1 and ZFP36L2 proteins in the effect of triciribine on *LDLR* mRNA stability. ZFP36L1 and ZFP36L2 destabilize *LDLR* mRNA in an ERK activity-sensitive manner^17^. Therefore, it can be envisioned that triciribine, through its ability to increase the activity of ERK, induces phosphorylation of ZFP36L1 and ZFP36L2, an event that is reported to result in increase in *LDLR* mRNA stability.

The involvement of ERK in regulation of *LDLR* mRNA stability and the dependence of triciribine on ERK activity to stabilize *LDLR* mRNA prompt us to put forward a simple mechanistic explanation for the stabilizing effect of triciribine on *LDLR* mRNA. AKT inhibits ERK activation by phosphorylating inhibitory sites in Raf, a kinase that functions as an upstream activator of ERK^52–54^. Inhibition of AKT by triciribine is then expected to alleviate the inhibitory effect of AKT on Raf, thus allowing downstream activation of ERK and the subsequent stabilization of LDLR mRNA.

Of importance is our finding that triciribine increases LDLR levels in both primary human hepatocytes and mouse liver. Therefore, we would like to suggest that triciribine represents a novel class of LDLR-inducing drugs with a therapeutic potential. The ability of triciribine to increase LDLR levels in cells that were cultured in either lipoprotein-containing medium or lipoprotein-deficient medium that were supplemented with statins (Fig. 4), a treatment reported to decrease cellular free cholesterol content^55^, suggests that triciribine is able to induce LDLR expression irrespective of cellular cholesterol status, raising the possibility that it can upregulate LDLR levels and consequently enhance cellular uptake of circulating cholesterol even under conditions where LDLR is inhibited by feedback inhibition. Furthermore, the ability of triciribine to enhance the effect of mevastatin on LDLR expression and cellular uptake of LDL suggests that it may also prove to exert an effective hypocholesterolemic effect when used in combination with statins. In this capacity, triciribine can not only be used to maximize the effect of statins, but it can also serve a statin dose-sparing function. Finally, we would like to note that given the involvement of AKT in a multitude of cellular events such as proliferation, apoptosis and insulin signaling^20^, it is justified to assume that inhibition of AKT by triciribine could carry a potential for adverse side effects. Therefore, we believe that prospective *in vivo* experiments should be carried out to determine whether triciribine, either alone or in combination with statins, evokes side effects which can negatively affect its potential as a hypocholesterolemic agent.

## Materials and Methods

### Reagents and antibodies

Triciribin and mevastatin were purchased from Selleckchem (Houston, Texas). Triciribine phosphate, U0126, actinomycin D (Act D), (±)-mevalonolactone, paraformaldehyde and saponin were from Sigma-Aldrich (St. Louis, Missouri). Antibodies against LDLR (3839; for Western blot analysis and immunofluorescence microscopy) and β-actin (ab8227) were obtained from BioVision (Milpitas, California) and Abcam (Cambridge, UK), respectively. Anti-LDLR (61087; for detection of cell-surface LDLR by flow cytometry) was obtained from Progen (Heidelberg, Germany). Anti-GAPDH (G9295) was from Sigma-Aldrich. Anti-β-tubulin (T9154-05G) was purchased from USBiological (Swampscott, Massachusetts).

### Cell culture and treatments

HepG2 cells (European Collection of Cell Cultures, Salisbury, UK), human telomerase reverse transcriptase (hTERT)-immortalized human hepatocytes (IHH; a gift from Dr. Philippe Collas, University of Oslo, Oslo, Norway) and HeLa cells (European Collection of Cell Cultures, Salisbury, UK) were cultured on collagen-coated culture vessel (BD Biosciences, San Jose, California) in HyClone Minimum Essential Medium (GE Healthcare Life Sciences, Pittsburg, Pennsylvania) supplemented with 10% fetal bovine serum (FBS; Sigma-Aldrich), 2 mM L-glutamine (Sigma-Aldrich), 50 U/mL penicillin (GE Healthcare Life Sciences), 50 µg/mL streptomycin (GE Healthcare Life Sciences) and non-essential amino acids (Biowest, Nuaillé, France). To obtain sterol-starved cells, cells were first cultured for 24 hours in complete medium supplemented with 10% FBS, washed twice in PBS and then cultured in complete medium containing 5% lipoprotein-deficient serum (LPDS). Freshly plated human hepatocytes were purchased from QPS Hepatic Biosciences (Research Triangle Park, North Carolina). Immediately on arrival, cells were provided with Maintenance Medium containing 0.1% dexamethasone (QPS Hepatic Biosciences) and incubated for 24 hour before treatment with triciribine. All cells were cultured at 37 °C in an atmosphere with 5% CO_2_ and 95% humidity. All drugs were dissolved in DMSO and added to cell cultures such that the final concentration of DMSO was kept at 0.1% (v/v). Control cultures were treated with DMSO alone at 0.1% (v/v).

### Western blot analysis

Cells were harvested by trypsinization and lysed inTriton X-100 lysis buffer (20 mM Tris [pH 7.5], 100 mM NaCl, 1% Triton X-100, 10 mM EDTA and Complete Protease Inhibitor Cocktail). After protein concentration determination using Pierce BCA protein assay (Thermo Fischer Scientific), equal amounts of proteins were resolved on a 4–20% SDS-PAGE, transferred to PDVF membrane (Bio-Rad, Hercules, California) and subjected to immunoblotting. After densitometric scanning of the immunoblots, the band intensity of the protein of interest was normalized to that of GAPDH or β-tubulin and plotted relative to the control value (vehicle-treated cells or cells harvested at 0 hour).

### Quantitative real-time PCR

Total RNA was isolated with the QIAamp RNA Isolation Kit (Qiagen, Hilden, Germany) and converted to cDNA using the AffinityScript QPCR cDNA Synthesis Kit (Agilent Technologies, Santa Clara, California). The cDNA was then used as the template for quantitative real-time polymerase chain reaction (qPCR) using Brilliant III Ultra-Fast QPCR Master Mix on Mx3005P QPCR system (Agilent technologies) and the following PrimeTime Predesigned qPCR Assays (Integrated DNA Technologies, Coralville, Iowa): human *LDLR* (Hs.PT.58.14599757), human *GAPDH* (Hs.PT.39a.22214836), human *HMGCR* (Hs.PT.58.41105492), human *PCSK9* (Hs.PT.58.203171419), *SREBP-2* (Hs.PT.56a.2651954) and human *TFRC* (Hs.PT.39a.22214826). qPCRs were run in duplicate and *GAPDH* was used as the normalizing gene. The 2^−ΔΔCt^ method was used to calculate relative mRNA levels.

### Analysis of cell-surface LDLR and LDL internalization

The levels of LDLR on the cell surface and internalization of LDL were measured by flow cytometry as previously described^56^.

### Analysis of LDLR expression by confocal fluorescence microscopy

Samples were fixed in 4% paraformaldehyde followed by permeabilization using 0.1% saponin. Cells were then incubated with anti-LDLR (1:100 dilution; BioVision) overnight. Alexa Fluor 488-conjugated anti-rabbit (1:200 dilution; 715–546–150 Jackson ImmunoResearch, Pennsylvania) was used for visualization. DAPI (ThermoFischer Scientific) was used to stain DNA. All pictures were taken with a Zeiss LSM 700 confocal system.

### Determination of PCSK9 in cell culture medium

The amount of PCSK9 secreted by cells into culture medium was measured using Circulex Human PCSK9 ELISA Kit (MBL International, Woburn, MA) according to the manufacturer’s instructions.

### DNA constructs, transfection and luciferase reporter assays

pLR1563-luc was kindly provided by Dr. Youngmi Kim Pak (Asian Institute for Life Sciences, University of Ulsan College of Medicine, Seoul, Korea)^29^. pLR1563/mutSRE-1-luc was generated by mutating CC (underlined) in the *LDLR* promoter SRE-1 motif (ATCACCCCAC) in pLR1563-luc to GG using QuickChange II XL mutagensis kit (Agilent Technologies). pmR-ZsGreen1-LDLR-3′UTR-wt was constructed by inserting a PCR fragment containing the 2.5 kbp of human *LDLR* 3′UTR downstream of *ZsGreen1* gene in pmR-ZsGreen1 reporter plasmid (Clonetech). For construction of pmR-ZsGreen1-LDLR-3′UTR-Del-ARE and pmR-ZsGreen1-LDLR-3′UTR-Del-IVS deletion mutants, we first used site-directed mutagenesis to generate unique restriction sites immediately upstream and downstream of the region to be deleted. The plasmids were then digested with the appropriate restriction enzymes and then religated to generate internally-deleted ZsGreen1-LDLR-3′UTR constructs. For construction of pmR-ZsGreen1-LDLR-3′UTR-Del-UCAU deletion mutant, we used PCR-mediated deletion technique to delete the region between ARE 1 and ARE 2 that encompasses the four UCAU tetranucleotide repeats. For transfection studies, HepG2 cells were transfected with 312 ng DNA/cm^2^ cells at a 4.5:1 FuGENE HD transfection reagent:DNA ratio according to the manufacturer’s instructions (Promega, Madison, Wisconsin). Transfection with empty vector served as control. Cells were treated with drugs at 24 hours after transfection. For quantification of ZsGreen1 fluorescence, cells were analyzed on a FACS Canto flow cytometer. To measure *LDLR* promoter activity, HepG2 cells were cotransfected with pRL-SV40 control vector and pLR1563-luc at a 1:10 ratio. Analysis of firefly and Renilla luciferase gene activities was performed by Dual-Luciferase Reporter Assay (Promega) following the manufacturer’s instructions.

### Triciribine *in vivo* activity

Six to eight week old male C57BL/6 J mice (Janvier Labs, Le Genest-Saint-Isle, France) were housed in a controlled environment with a 12 hour light/dark cycle at 22 °C. All mice were acclimatized for 7 days before a 2-weeks treatment period and were fed a normal chow diet (801700; SDS, Essex, UK) during both acclimatization and treatment periods. Mice were separated into three treatment groups each consisting of 4 mice. One group received i.p. triciribine at 1 mg/kg daily. A second group received daily i.p. injections of triciribine at 2 mg/kg. A third group was administered daily i.p. injections of vehicle (0.1% DMSO in PBS). Injection volumes were 100 µl per mouse. At the end of the treatment period, mice were sacrificed and livers were processed into a powder by grinding in liquid nitrogen, lysed in RIPA buffer and then analyzed by Western blotting. Experiments were performed in accordance with institutional guidelines and were approved by The Norwegian Animal Research Authority.

### Statistical analysis

The difference between the means of two independent groups was examined by unpaired, two-tailed Student’s *t*-test and a *p* value of <0.05 was considered statistically significant.

## Electronic supplementary material

Supplementary information
