# Supplementary material for: Triciribine increases LDLR expression and LDL uptake through stabilization of LDLR mRNA
Source: Sci Rep. 2018 Nov 1;8:16174. doi: 10.1038/s41598-018-34237-6 (PMC6212527; doi:10.1038/s41598-018-34237-6)
Supplement: Supplementary file 1 — Supplementary information [file 41598_2018_34237_MOESM1_ESM.docx]

**Triciribine increases LDLR expression and LDL uptake through stabilization of *LDLR* mRNA**

Katrine Bjune, Lene Wierød and Soheil Naderi^*^

Unit for Cardiac and Cardiovascular Genetics, Department of Medical Genetics, Oslo University Hospital, Oslo, Norway

**^*^Corresponding author.** Unit for Cardiac and Cardiovascular Genetics, Department of Medical Genetics, Oslo University Hospital, Oslo, Norway

e-mail: [sohnad@ous-hf.no](mailto:sohnad@ous-hf.no)

**Supplementary materials and methods**

**Reagents and antibodies**

DTT, 25-hydroxycholesterol (25-HC) and 5-Azacytidine (5-AzaC) were obtained from Sigma-Aldrich (St. Louis, Missouri). GSK690693 was obtained from Selleckchem (Houston, Texas). Anti-phospho-AKT (pAKT, S473; AF887) and anti-AKT (MAB2055) antibodies were from R&D Systems (Minneapolis, MN). Antibodies raised against the C-terminus of SREBP-2 (557037) were purchased from BD Biosciences (San Jose, CA). Anti-calreticulin (12238), anti-IRE1α (3294) and anti-phospho-GSK-3β (pGSK-3β, S9) were from Cell Signaling (Danvers, MA).

**Determination of *XBP1* splicing**

Total RNA (1 µg) isolated from CHO cells was reverse-transcribed with the Qiagen Onestep RT-PCR kit (Qiagen, Hilden, Germany) and the following X-Box Binding Protein 1 (*XBP1*)-specific primers^1^; forward primer: 5’-CCTTGTAGTTGAGAACCAGG-3’; reverse primer: 5’- GGGGCTTGGTATATATGTGG-3’. The reaction conditions consisted of a 15-minute polymerase activation step at 95°C followed by 40 tri-step cycles of denaturation at 95°C for 1 minute, annealing at 60°C for 1 minute and extension at 72°C for 1 minute. The final PCR products were then separated on a 2% agarose gel containing GelRed Nucleic Acid Gel Stain (Biotium, Fermont, CA) along with DNA markers.

**Cell fractionation**

HepG2 cells were harvested by trypsinization and pellets were washed in PBS before incubation in hypotonic buffer (10 mM Tris [pH 7.6], 1.5 mM MgCl2, 10 mM KCl, 0.5 mM DTT) for 15 minutes on ice. Cells were then lysed by 25 passages through a 25-gauge needle and centrifuged at 1000x*g* for 10 min at 4°C to collect the nuclei. The nuclei pellet was resuspended in 100 μl RIPA buffer (50 mM Tris [pH 7.5], 150 mM NaCl, 1% NP-40, 0.1% SDS, 0.5 mM EDTA, 10 mM NaF, 5 mM β-glycerophosphate, 0.1 mM Na3VO4 and Complete Protease Inhibitor Cocktail [Sigma-Aldrich]) and the suspension was incubated on ice for 40 minutes with intermittent vortexing at 2000 rpm and then centrifuged at 20,000x*g* for 30 minutes at 4°C. The recovered supernatant was designated as nuclear extract. The supernatant recovered from the 1000x*g* centrifugation was spun at 20,000x*g* for 30 minutes at 4°C to pellet membranes. The membrane pellet was then resuspended in 100 μl RIPA and incubated on ice for 40 minutes with intermittent vortexing at 2000 rpm to extract membrane proteins and then clarified by centrifugation at 20,000x*g* for 30 minutes at 4°C. The supernatant was designated as the membrane fraction.

**Supplementary figures and figure legends**


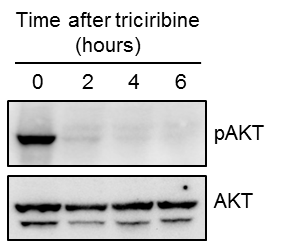


**Supplementary Figure S1. Effect of triciribine on AKT phosphorylation.** Sterol-fed HepG2 cells were treated with or without 1 µM triciribine for the indicated times before harvesting for analysis by immunoblotting with the antibodies against phospho-AKT (pAKT) and total AKT. One representative blot of three is shown. Unprocessed blots are shown in Supplementary Fig. S16.


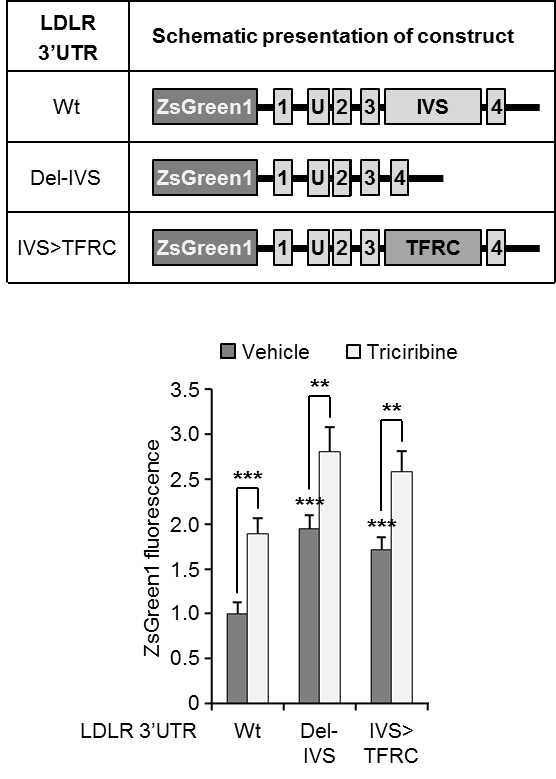


**Supplementary Figure S2. Effect of IVS deletion on *LDLR* mRNA.** Upper panel: schematic presentation of the chimeric ZsGreen1-LDLR-3’UTR constructs. Lower panel: at 24 hours after transfection of sterol-fed HepG2 cells with one of the constructs, cells were treated with 1 µM triciribine and then harvested for flow cytometric analysis of mean ZsGreen1 fluorescence intensity as an indicator of *ZsGreen1* transcript levels. Results were then plotted relative to vehicle-treated cells that were transfected with ZsGreen1-LDLR-3’UTR-wt construct (n = 4). Error bars represent SD. ^★★^*p* < 0.01 and ^★★★^*p* < 0.001 compared with matched vehicle-treated cells. AREs are represented with numerically labelled rectangles. Letter U refers to UCAU repeats. IVS, intervening sequence; TFRC, transferrin receptor.


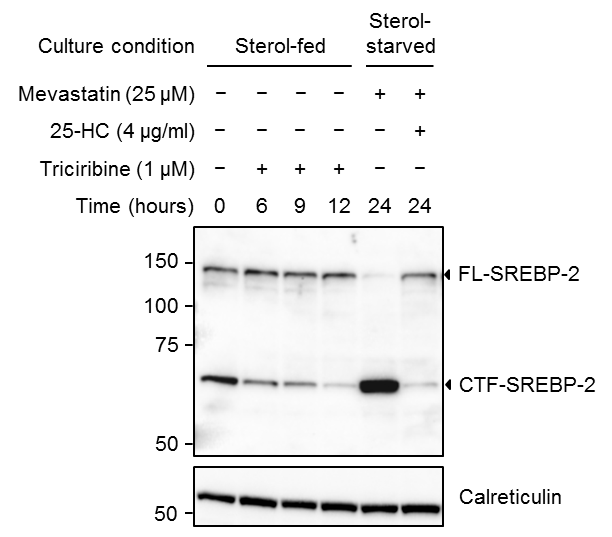


**Supplementary Figure S3. Effect of triciribine on levels of the C-terminal fragment of SREBP-2.** Sterol-fed HepG2 cells were treated with 1 µM triciribine for the indicated times before harvesting. Sterol-starved cells that were treated with mevastatin or mevastatin + 25-hydroxycholesterol (25-HC) served as controls for induction and inhibition of SREBP-2 cleavage, respectively. Cells were then subjected to subcellular fractionation before Western blot analysis for expression of SREBP-2 and calreticulin. The blot shows one of three independent experiments. FL-SREBP-2, full-length SREBP-2; CTF-SREBP-2, C-terminal fragment of SREBP-2. Unprocessed blots are shown in Supplementary Fig. S17.


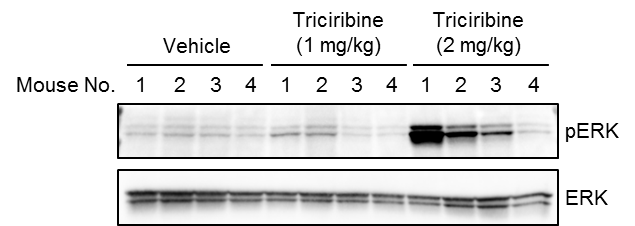


**Supplementary Figure S4. Effect of triciribine on ERK phosphorylation in mouse liver.** Equal amount of mouse liver lysates (50 µg) for the experiment of Fig. 7b were run on duplicate gels and transferred to PVDF membrane. One membrane was immunoblotted with anti-phospho-ERK (pERK) and the duplicate membrane was immunoblotted with anti-total ERK. Unprocessed blots are shown in Supplementary Fig. S18.

**
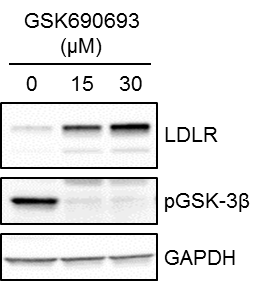
**

**Supplementary Figure S5. Induction of LDLR levels by GSK690693.** Sterol-fed HepG2 cells were treated with the indicated concentrations of GSK690693 for 14 hours and then harvested for immunoblot analysis with antibodies against LDLR, phospho-GSK-3β (pGSK-3β) and GAPDH. One representative experiment of three is shown. Unprocessed blots are shown in Supplementary Fig. S19.


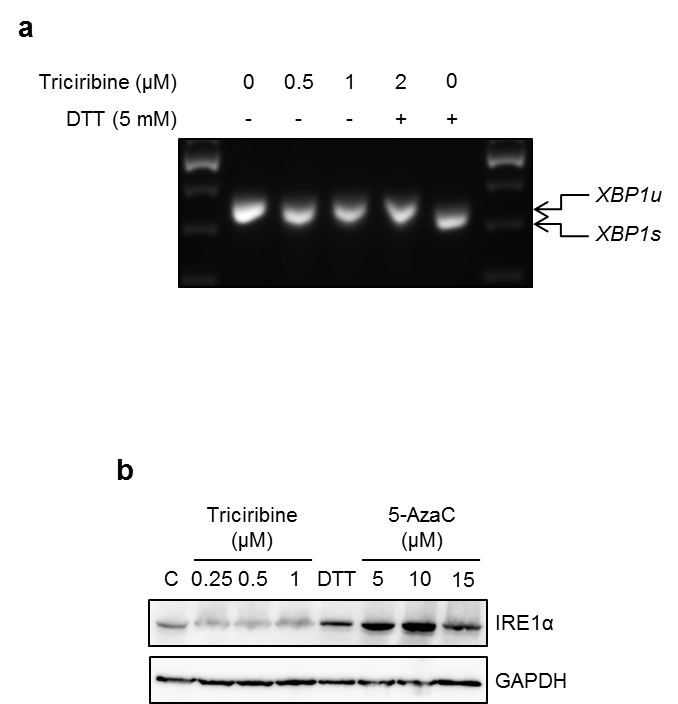


**Supplementary Figure S6. Effect of triciribine on expression of ER stress markers.** (a) Sterol-fed HepG2 cells were treated with vehicle or triciribine for 14 h. As a positive control for ER stress, cells were treated with DTT for 14 hours. Total RNA was isolated, subjected to RT-PCR with *XBP1*-specific primers and the PCR products were then resolved on 2% agarose gel for separation of unspliced *XBP1* (*XBP1u*) and spliced *XBP1* (*XBP1s*) mRNAs. One representative experiment of two is shown. Unprocessed image is shown in Supplementary Fig. S20. (b) Sterol-fed HepG2 cells were treated with the indicated concentrations of triciribine or 5-AzaC (5-Azacytidine). Cells treated with 5 mM DTT served as positive control for expression of IRE1α. At 16 hours post-treatment, cells were harvested and subjected to immunoblotting with the indicated antibodies. One representative blot of three independent experiments is shown. Unprocessed blots are shown in Supplementary Fig. S21.

**
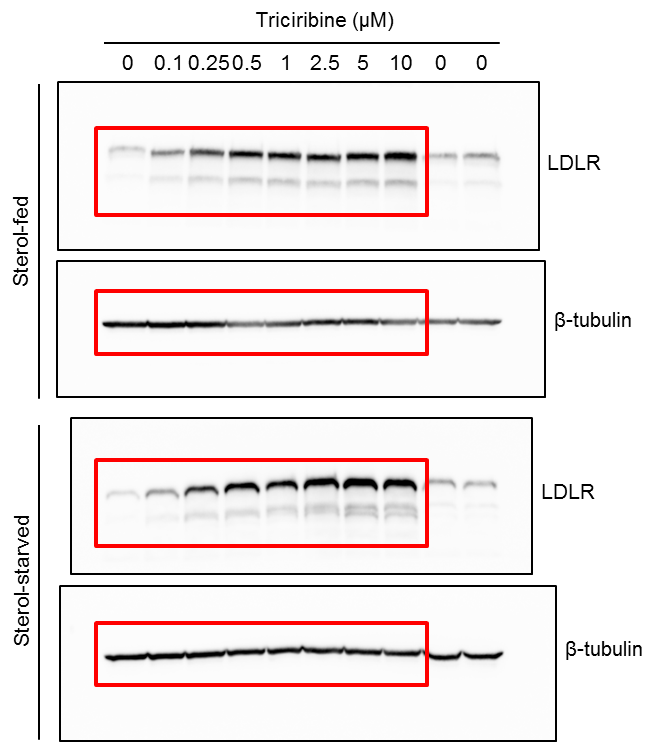
**

**Supplementary Figure S7.** Unprocessed blots used in Fig. 1a.

**
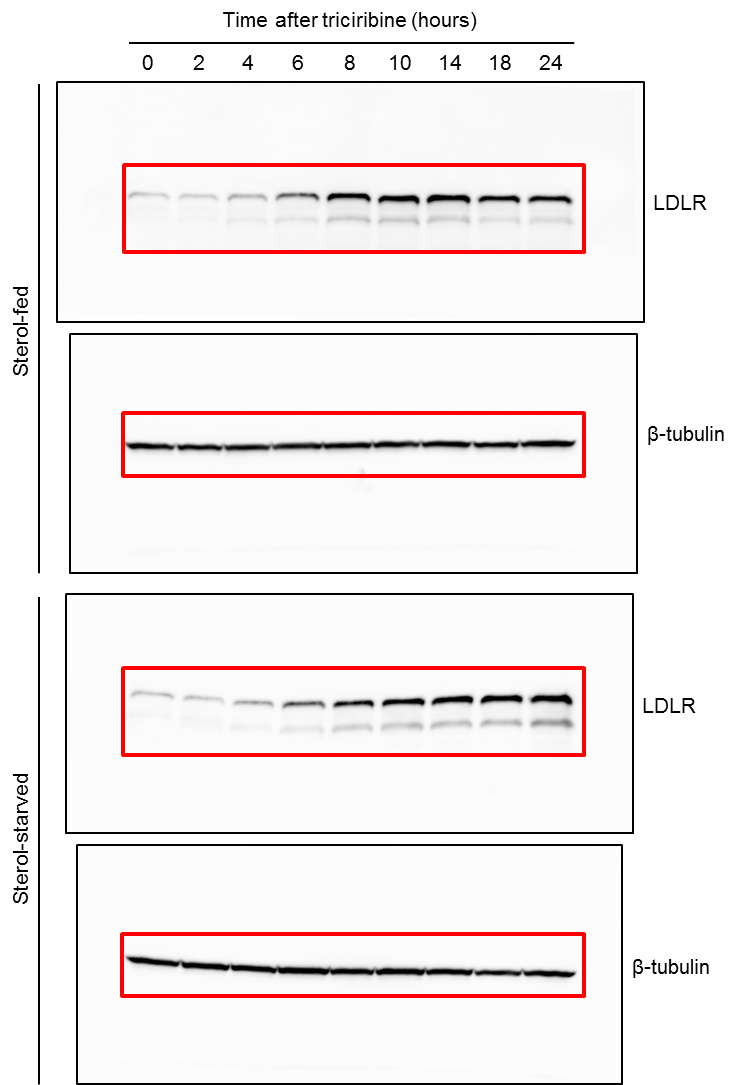
**

**Supplementary Figure S8.** Unprocessed blots used in Fig. 1b.

**
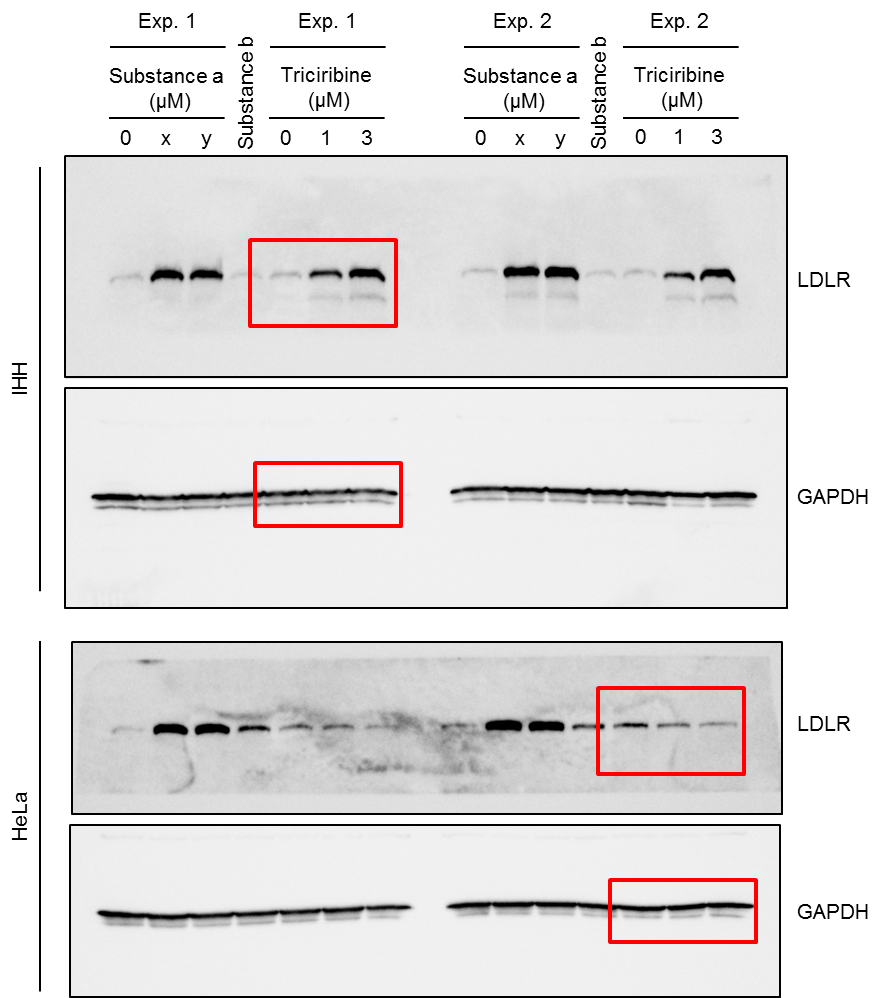
**

**Supplementary Figure S9.** Unprocessed blots used in Fig. 1f.

**
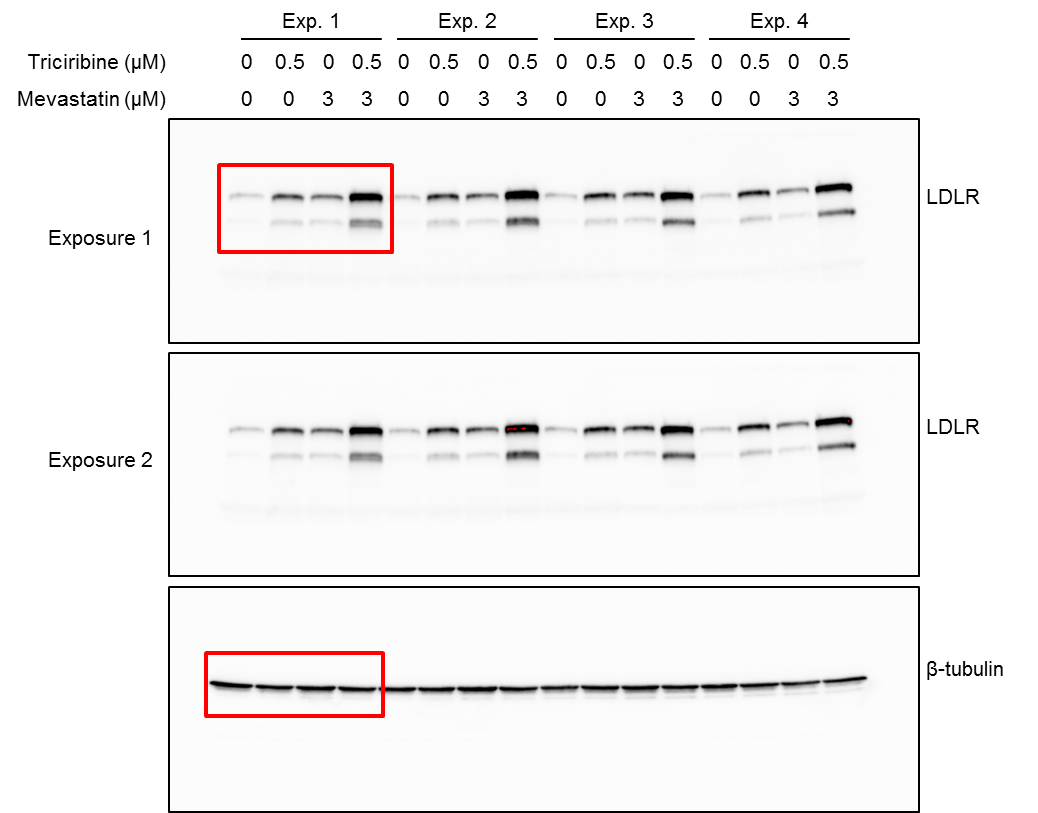
**

**Supplementary Figure S10.** Unprocessed blots used in Fig. 4a.

**
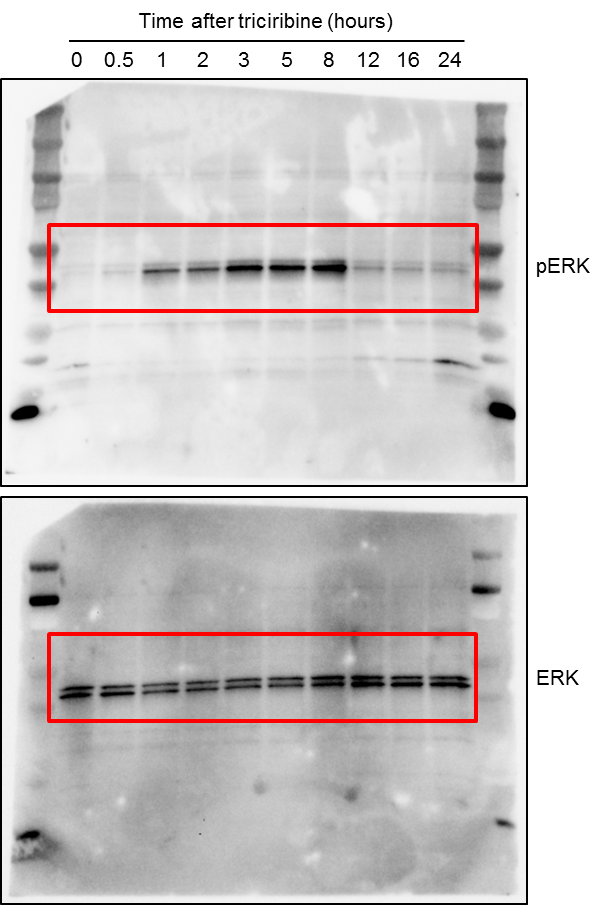
**

**Supplementary Figure S11.** Unprocessed blots used in Fig. 5a.

**
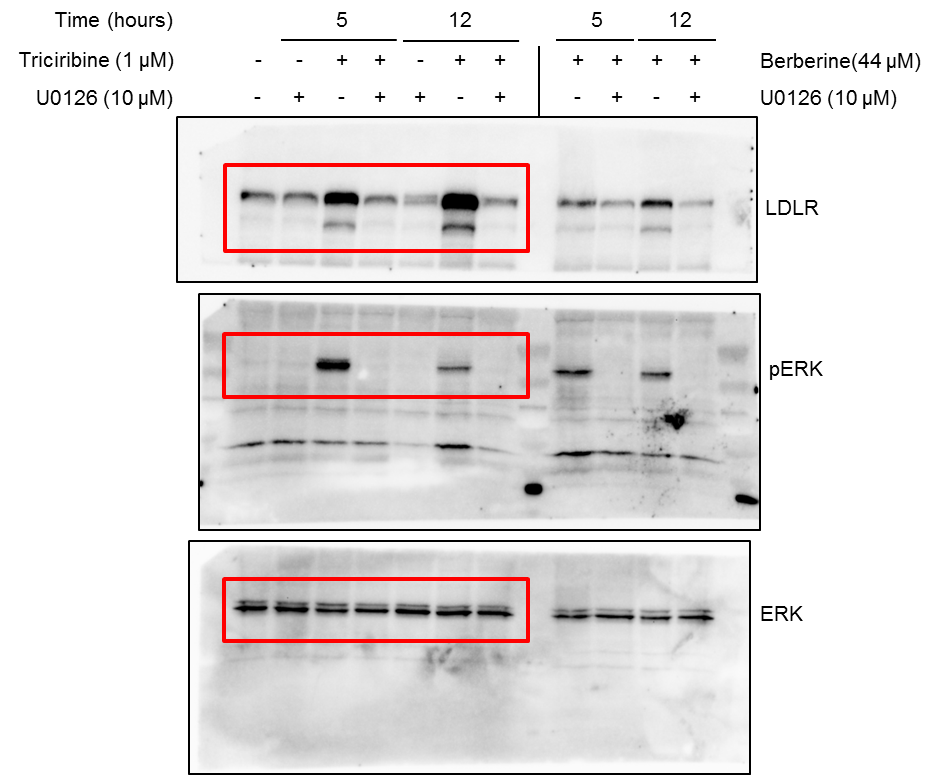
**

**Supplementary Figure S12.** Unprocessed blots used in Fig. 5b.

**
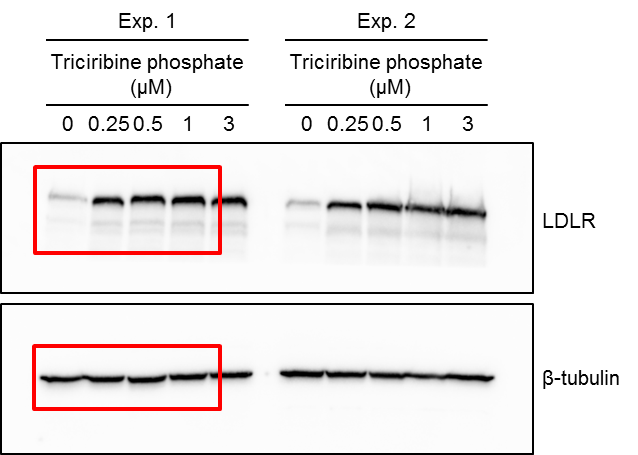
**

**Supplementary Figure S13.** Unprocessed blots used in Fig. 6a.

**
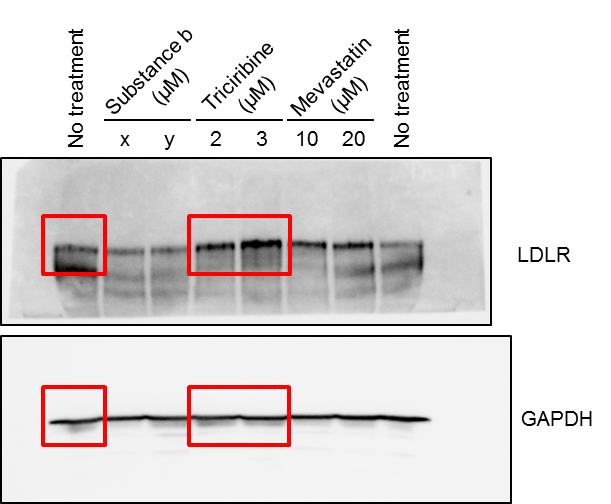
**

**Supplementary Figure S14.** Unprocessed blots used in Fig. 7a.

**
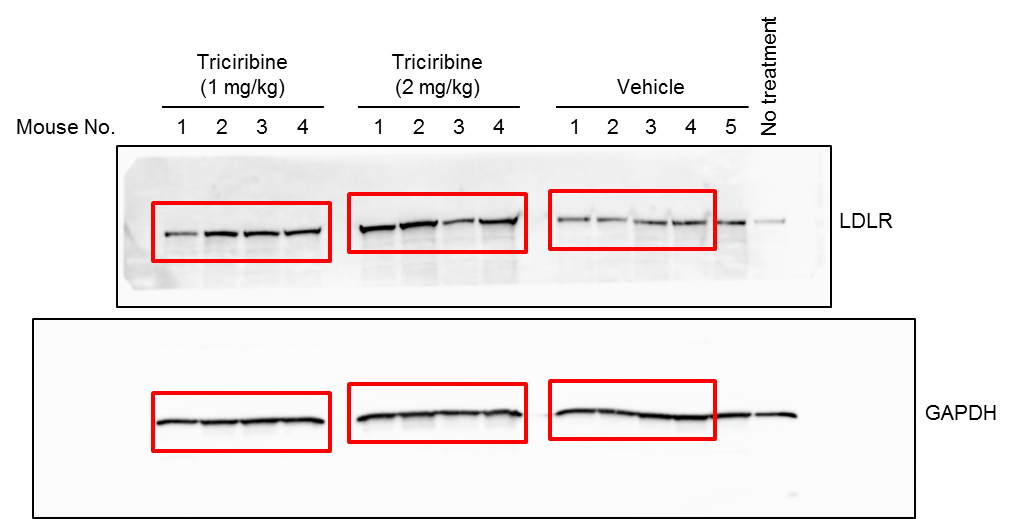
**

**Supplementary Figure S15.** Unprocessed blots used in Fig. 7b.

**
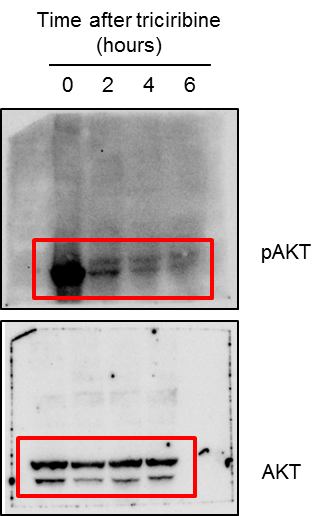
**

**Supplementary Figure S16.** Unprocessed blots used in Supplementary Fig. S1.

**
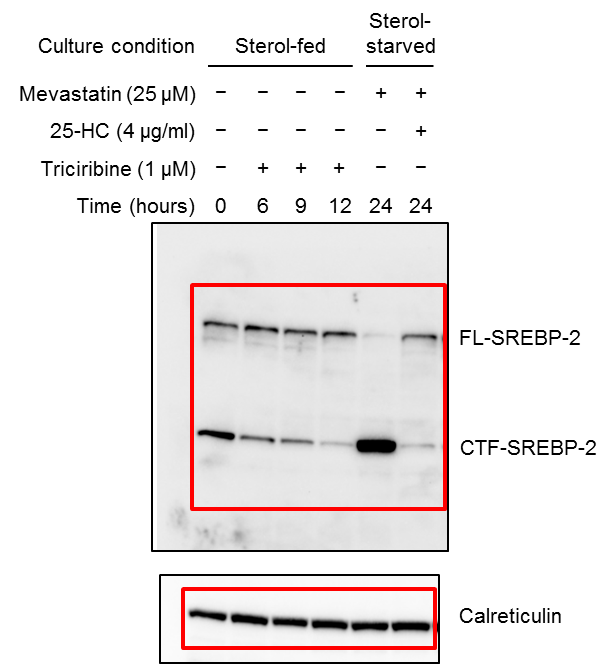
**

**Supplementary Figure S17.** Unprocessed blots used in Supplementary Fig. S3.

**
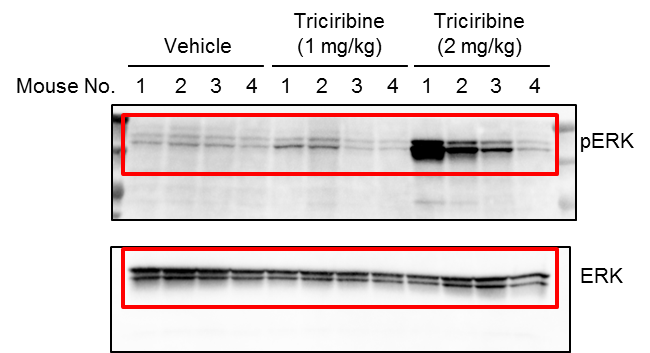
**

**Supplementary Figure S18.** Unprocessed blots used in Supplementary Fig. S4.

**
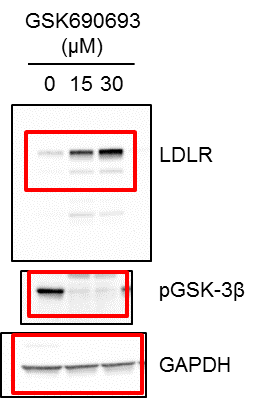
**

**Supplementary Figure S19.** Unprocessed blots used in Supplementary Fig. S5.

**
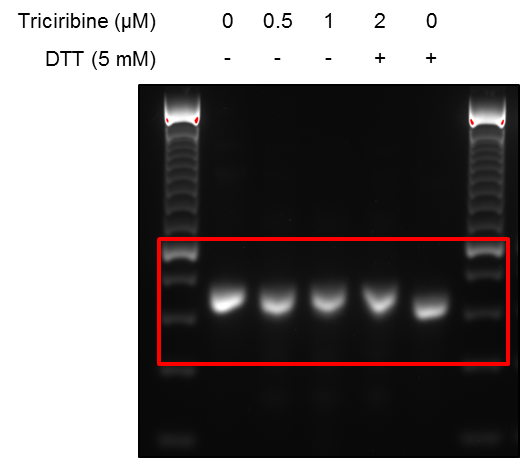
**

**Supplementary Figure S20.** Unprocessed image used in Supplementary Fig. S6a.

**
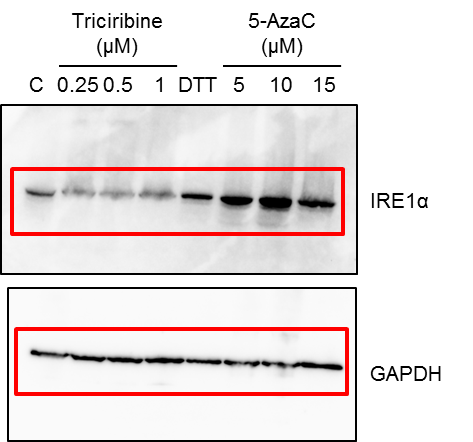
**

**Supplementary Figure S21.** Unprocessed blots used in Supplementary Fig. S6b.

**REFERENCES**

1 Szczesna-Skorupa, E., Chen, C. D., Liu, H. & Kemper, B. Gene expression changes associated with the endoplasmic reticulum stress response induced by microsomal cytochrome p450 overproduction. *The Journal of biological chemistry* **279**, 13953-13961 (2004).
